# Untargeted muscle tissue metabolites profiling in young, adult, and old rats supplemented with tocotrienol-rich fraction

**DOI:** 10.3389/fmolb.2022.1008908

**Published:** 2022-10-14

**Authors:** Siti Liyana Saud Gany, Jen Kit Tan, Kok Yong Chin, Nur Haleeda Hakimi, Nazirah Ab Rani, Nurhazirah Ihsan, Suzana Makpol

**Affiliations:** ^1^ Department of Biochemistry, Faculty of Medicine, Universiti Kebangsaan Malaysia Medical Centre, Kuala Lumpur, Malaysia; ^2^ Department of Pharmacology, Faculty of Medicine, Universiti Kebangsaan Malaysia Medical Centre, Kuala Lumpur, Malaysia; ^3^ Menara Sime Darby Oasis Corporate Park, Petaling Jaya, Malaysia

**Keywords:** tocotrienol, sarcopenia, untargeted metabolites, skeletal muscle, ageing

## Abstract

The greatest significant influence on human life span and health is inevitable ageing. One of the distinguishing characteristics of ageing is the gradual decrease of muscle mass and physical function. There has been growing evidence that tocotrienol can guard against age-associated chronic diseases and metabolic disorders. This study aimed to elucidate the effects of tocotrienol-rich fraction (TRF) on muscle metabolomes and metabolic pathways in ageing Sprague Dawley (SD) rats. Three months, 9 months, and 21 months old male SD rats were divided into control and treated groups with 10 rats per group. Rats in control and treated groups were given 60 mg/kg body weight/day of palm olein and 60 mg/kg body weight/day of TRF, respectively, *via* oral gavage for 3 months. Muscle performance was assessed at 0 and 3 months of treatment by measuring muscle strength and function. Our results showed that TRF treatment caused a significant increase in the swimming time of the young rats. Comparison in the control groups showed that metabolites involved in lipid metabolisms such as L-palmitoyl carnitine and decanoyl carnitine were increased in ageing. In contrast, several metabolites, such as 3-phosphoglyceric acid, aspartic acid and aspartyl phenylalanine were decreased. These findings indicated that muscle metabolomes involved in lipid metabolism were upregulated in aged rats. In contrast, the metabolites involved in energy and amino acid metabolism were significantly downregulated. Comparison in the TRF-supplemented groups showed an upregulation of metabolites involved in energy and amino acid metabolism. Metabolites such as N6-methyl adenosine, spermine, phenylalanine, tryptophan, aspartic acid, histidine, and N-acetyl neuraminic acid were up-regulated, indicating promotion of amino acid synthesis and muscle regeneration. Energy metabolism was also improved in adult and old rats with TRF supplementation as indicated by the upregulation of nicotinamide adenine dinucleotide and glycerol 3-phosphate compared to the control group. In conclusion, the mechanism underlying the changes in skeletal muscle mass and functions in ageing was related to carbohydrate, lipid and amino acid metabolism. Tocotrienol supplementation showed beneficial effects in alleviating energy and amino acid synthesis that may promote the regeneration and renewal of skeletal muscle in ageing rats.

## 1 Introduction

The greatest significant influence on human life span and health is inevitable ageing. Most individuals on earth are middle-aged or older, and in high-income nations, the proportion of those over 60 is increasing faster than that of any other age group (Beard et al., 2016). The number of Americans 65 years old and above was anticipated to be around 43.1 million in 2012, and by the year 2050, it is expected to increase to 83.7 million (Ortman et al., 2014). By 2050, 1.6 billion people over 65 are expected to live on the planet (Roberts et al., 2018). This demographic transformation is one of the most significant socio-economic issues of the current decade because ageing is linked to several comorbidities that lead to fast-rising health care expenses (Gasior et al., 2012). One of the distinguishing characteristics of ageing is the gradual decrease of muscle mass and physical function, often known as sarcopenia (Roubenoff, 2000). Sarcopenia typically begins in the fifth decade of life (Miljkovic et al., 2015; Cruz-Jentoft et al., 2010; Clark and Manini, 2012). It can affect mobility and increase the risk of falling, thus increasing the cost of healthcare and hospitalisation, which increases the economic burden within the society (Chung et al., 2018). Since sarcopenia is now recognised as a significant clinical phenomenon, interdisciplinary efforts are being made to recognise, comprehend, prevent and treat this disorder (Cooper et al., 2013; Jean-Yves Reginster, 2016).

Insight into the molecular mechanisms that cause ageing has advanced remarkably. Sarcopenia may result from various age-related molecular and cellular damage, also known as the hallmarks or pillars of ageing. These include loss of proteostasis, dysregulated nutrient sensing, stem cell exhaustion/dysfunction, cellular senescence, genomic instability, mitochondrial dysfunction and epigenetic alterations (López-Otín et al., 2013) (Kennedy et al., 2014). One of the most significant roles of skeletal muscle is its exceptional ability to produce force and power. Strength, or the maximum capacity of skeletal muscle to generate force, is a function of the muscle’s cross-sectional area and the nervous system’s ability to activate the corresponding motor fully. According to cross-sectional research, older adults strongly correlate muscle size and maximum strength. However, a more nuanced association has been found in studies that tracked changes in muscle strength and mass over time (Goodpaster et al., 2006) (Lauretani et al., 2003).

The simultaneous decrease of skeletal muscle mass and strength with age affects independence and quality of life by reducing locomotor performance and jeopardising metabolic health. This is shown in the solid epidemiological links between sarcopenia and dynapenia, risk of non-communicable diseases (such as diabetes, chronic obstructive pulmonary disease and cancer), and ensuing morbidity and mortality (Nichols et al., 2019; Newman et al., 2006). Developing methods for stratification, identifying targets for treatment, and triaging those in need of early intervention could all be accomplished by identifying molecular signals linked to muscle ageing (Sood et al., 2015; Xia et al., 2013).

In recent years, utilising omics technologies (such as transcriptomics, proteomics and metabolomics) has allowed researchers to better understand the underlying causes of various illness states and create biomarkers for either diagnosis or prognosis (Bhattacharya et al., 2014; Zierer et al., 2015). For instance, metabolomic methods have already provided insight into alterations in the muscle metabolome with ageing. A recent study by Fazelzadeh and others into age-related changes in muscle metabolome revealed that metabolites linked to mitochondrial function, fibre type and tissue turnover varied between age groups (Fazelzadeh et al., 2016). It is anticipated that local levels of metabolites will reflect the age-related changes in gene expression levels in muscle. According to a recent animal study, age impacts muscle glucose and fatty acid metabolism (Houtkooper et al., 2011). In another study, it was found that ageing causes changes in lipid and glucose metabolism that are specific to certain muscle groups and are consistent with mitochondrial dysfunction (Garvey et al., 2014).

Intriguingly, baseline levels of metabolites from the tricarboxylic acid cycle were reported to be lower in healthy older adults than in younger people in muscle biopsies. These differences were accompanied by lower concentrations of ATP, ADP, branched-chain amino acids, and acylcarnitine in older, healthy subjects, suggesting impaired mitochondrial function or a lower number of mitochondria in the older subjects’ muscle, which in turn may be a result of the older subjects’ lower habitual physical activity (Fazelzadeh et al., 2016). Biochemical features of mammals’ skeletal muscle have been shown to change in the course of ageing, mainly within the energy metabolism system (Ermini 1976). This study looks into untargeted metabolomics whereby all the low molecular weight compounds or metabolites that are present and participate in biochemical reactions at a particular time are captured and quantified (Horgan and Kenny 2011). Utilising a metabolomic-based approach could provide insight into the overall molecular mechanisms of disease progressions and how a therapeutic agent works at a metabolic level.

The metabolic basis of sarcopenia is not very much known, and there have been no reports on how tocotrienol can affect the metabolic profiling of skeletal muscle in ageing and sarcopenia. Sarcopenia has been shown to decrease metabolic activities in mitochondria, muscle, kidney, and methylation compared with the declined metabolic activities for antioxidation in frailty (Kameda et al., 2020). There has been growing evidence that tocotrienol, which is a vitamin E isomer, can prevent the onset of age-related chronic illnesses and metabolic disturbances, like obesity (Zhao et al., 2016; Fukui 2019; Pang and Chin 2019), cardiovascular disease (Ramanathan et al., 2018), type-2 diabetes mellitus (Chung et al., 2020; Mahjabeen et al., 2021), arthritis (Haleagrahara et al., 2014; Radhakrishnan et al., 2014; Chin et al., 2019), osteoporosis (Shen et al., 2017) and sarcopenia (Khor et al., 2014; Khor et al., 2016; Chung et al., 2018; Lim et al., 2019).

Vitamin E comprises two subgroups: tocopherols and tocotrienols (Schneider 2005). It has been established that tocotrienols have superior anti-inflammatory and antioxidant activities compared to α-tocopherols (Peh et al., 2016). Palm oil is the most abundant source of tocotrienols, referred to as tocotrienol-rich fraction (TRF). Using palm oil as a lipid carrier appears to enhance the bioavailability and solubility of the treatment, improving the permeability and effectiveness of medications, stabilising the emulsification of formulation between emulsifier and surfactant, and extending the shelf life of the therapeutic agent. Palm oil has demonstrated beneficial behaviour in giving variety in medicine design, shape and delivery (Goon et al., 2019). The long-chain triglycerides in palm oil are enzymatically processed before being taken up by enterocytes and absorbed into the bloodstream (Goon et al., 2019).

The skeletal muscle ageing process is typically accompanied by impaired muscle metabolism, including mitochondrial dysfunction and insulin resistance (Distefano and Goodpaster, 2017). However, strategies to slow ageing have mostly failed to achieve their desired response. As a result, the current study aimed to identify any potential impacts of TRF on several metabolic pathways in the ageing Sprague Dawley rat’s muscle tissue.

## 2 Materials and methods

### 2.1 Animal model

Three months old, 9 months old and 21 months old male Sprague Dawley (SD) rats were purchased from Laboratory Animal Resources Unit (LARU), University Kebangsaan Malaysia. They were separated into three groups of young (3 months old), adult (9 months old) and old (21 months old). Rats in each group were further divided into subgroups of control and treated groups with 10 rats per group. After a week of acclimatisation, rats were housed one per cage and provided food and water *ad libitum*. Rats in control and treated groups were given 60 mg/kg body weight/day of refined, bleached, and deodorised (RBD) palm olein (Sime-Darby Plantation Berhad) and 60 mg/kg body weight/day of tocotrienol-rich fraction (TRF) (Sime-Darby Plantation Berhad) *via* oral gavage for 3 months respectively. Muscle function tests were performed at 0 months before the start of the TRF administration and after 3 months of TRF administration. Blood samples were also taken *via* retro-orbital sinus at 0 months, 1.5 and 3 months of TRF administration. After 3 months of the TRF administration, the rats were sacrificed, and organs were harvested, including the gastrocnemius and soleus muscle from both hind legs. They were snap frozen and stored at −80°C until further analyses. The University Kebangsaan Malaysia ethical committee approved all animal protocols used in this study with approval number BIOK/FP/2020/SUZANA/25-MAR/1099-MAR-2020-DEC.2022.

### 2.2 Vitamin E preparation

TRF (Golden Tri™ E 70) and RBD palm olein were a gift from Sime Darby Plantation Berhad. TRF consists of 24% α-tocopherol, 27% α-tocotrienol, 4% β-tocotrienol, 32% γ-tocotrienol and 14% δ-tocotrienol in every Gram of TRF. RBD palm olein consist of 0.054% free fatty acids, 0.043% moisture and impurities, 0.45% peroxide values and 64.71% iodine value. 60 mg/kg body weight of TRF was administered to the rats in the treated group over 3 months. TRF mixture was prepared every week in a dark room by adding 2.4 g of TRF into 40 ml of RBD palm olein in a falcon tube and vortexed until thoroughly mixed. The falcon tube was wrapped with aluminium foil to protect it from sunlight and kept at 4°C.

### 2.3 Muscle function tests

#### 2.3.1 Weight-loaded performance swim test

Experimental rats were loaded with the weight of 3% body weight at the base of the tail and forced to swim in the water of 22 ± 1°C temperature and 30 cm depth. The time from when the rats were put in the water to the point at which rats failed to return to the water surface within 10 s was recorded as exhaustion time.

### 2.3.2 Grid-hang test

The rat was placed onto the cage lid. The rat was allowed to accommodate to the environment for 3–5 s before the lid was turned over slowly and held at least 30 cm over a rat cage containing 5–7 cm soft bedding. The cage lid hang time (seconds) is defined as the amount of time it takes the rat to fall from the inverted lid. The hang time was measured from the time the lid was inverted to the time that the rat fell off the lid (determined visually and measured using a stopwatch). The procedure was repeated three times with a rest interval between hang attempts of 2–3 min. The “Holding Impulse” which is associated with the hanging test is equivalent to the hang time multiplied by the body weight (gm sec or Newtons sec; with a conversion factor—9.806 × 10^–3^ N/gm).

### 2.4 Euthanization of animals

On day 90 of the study, all rats were fasted for the night before being sacrificed for necropsy analysis. The combination of ketamine, xylazine and zoletil-50 (tiletamine and zolazepam) known as KTX agents was utilised as the anaesthetic in this study. The lower right quadrant of the abdomen was chosen for intraperitoneal injection of the KTX agents because of their speed, effectiveness, and lack of discomfort. Each rat received 0.1 ml/250 g of body weight KTX. The rats were then left for about 30 min to allow the KTX agents to give their sedative effects. This was observed by clinical indicators which include confusion and loss of consciousness, depression of respiration or rapid, irregular breathing, steadily declining heart rate and blood pressure, as well as urine and defecation. The rats were afterwards euthanised by decapitation using a decapitator from Modiezhan Sdn. Bhd, Kuala Lumpur, Malaysia.

### 2.5 Collection of organs

The sacrificed rats’ internal organs, including the brain, heart, lungs, liver, spleen, kidneys, gastrocnemius muscle and soleus muscle were dissected. The weight of the organs was taken as promptly as possible to prevent drying, and they were examined in relation to the body weight of the animals after being washed with 90% normal saline to remove any adhering tissue. The organs were then kept frozen in a -80°C freezer.

### 2.6 Tissue sample preparation

Gastrocnemius muscle was thawed, and 1 g of tissue was dissected. Samples were cut into smaller pieces using scissors and transferred to a 15 ml tube. 1 ml of mass spec water was added into the tube and the samples were sonicated in ice. Samples were kept homogenated at -80°C until ready to be used.

### 2.7 Metabolomic analysis by UHPLC-MS/MS

A muscle tissue sample was thawed and placed in a glass tube filled with one ul Avanti (internal standard), 800 ul 100% methanol (MeOH) and subsequently vortexed. Then, 1.6 ml 100% Dicromethane (DCM) was added using a glass pipette and incubated in a vortex at room temperature for 1 hour at 300 rpm. After that, 600 ul of distilled water was added to form two layers. The sample was then vortexed for 1,000 × g for 10 min at room temperature. Then 1.0 ml lower phase (organic/lipid) was collected in a new glass tube and dried at 1,000 rpm at room temperature in a centrifuge vacuum. Then 1.0 ml upper phase (aqueous/polar/metabolomic) was collected and dried. The dried extracts were kept at −80°C. When the samples were ready to run, it was reconstituted with 100 ul isopropanol:MeOH (1:1) and 100 ul was transferred through a filter to vials using syringe and syringe filters. For the quality control (QC) sample, 5 uL of all samples were mixed from each vial into a tube, spun down, and 100 ul were transferred into a vial and capped (Goon et al., 2021). The vials were randomly placed into the Liquid Chromatography-Mass Spectrometry and run for 3 days. Untargeted metabolomics analysis was performed using the UHPLC system (Dionex Ultimate 3,000; Thermo specific) and Orbitrap-MS (Q Exactive HF, Thermo Scientific).

### 2.8 Statistical analysis

Metabolomic profiles were compared among groups using MetaboAnalyst 5.0 software. Principal component analysis (PCA) was used to perform multivariate data analysis. Data sets were then scaled and processed to minimise technical variability between individual samples before extracting pertinent biological information (van den Berg et al., 2006). T-tests and fold-change analysis were conducted (Xia and Wishart 2016). A *p*-value of 0.05 was regarded as statistically significant.

## 3 Result

### 3.1 Measurement of exhaustion time *via* weight-loaded performance swim test

**FIGURE 1:**
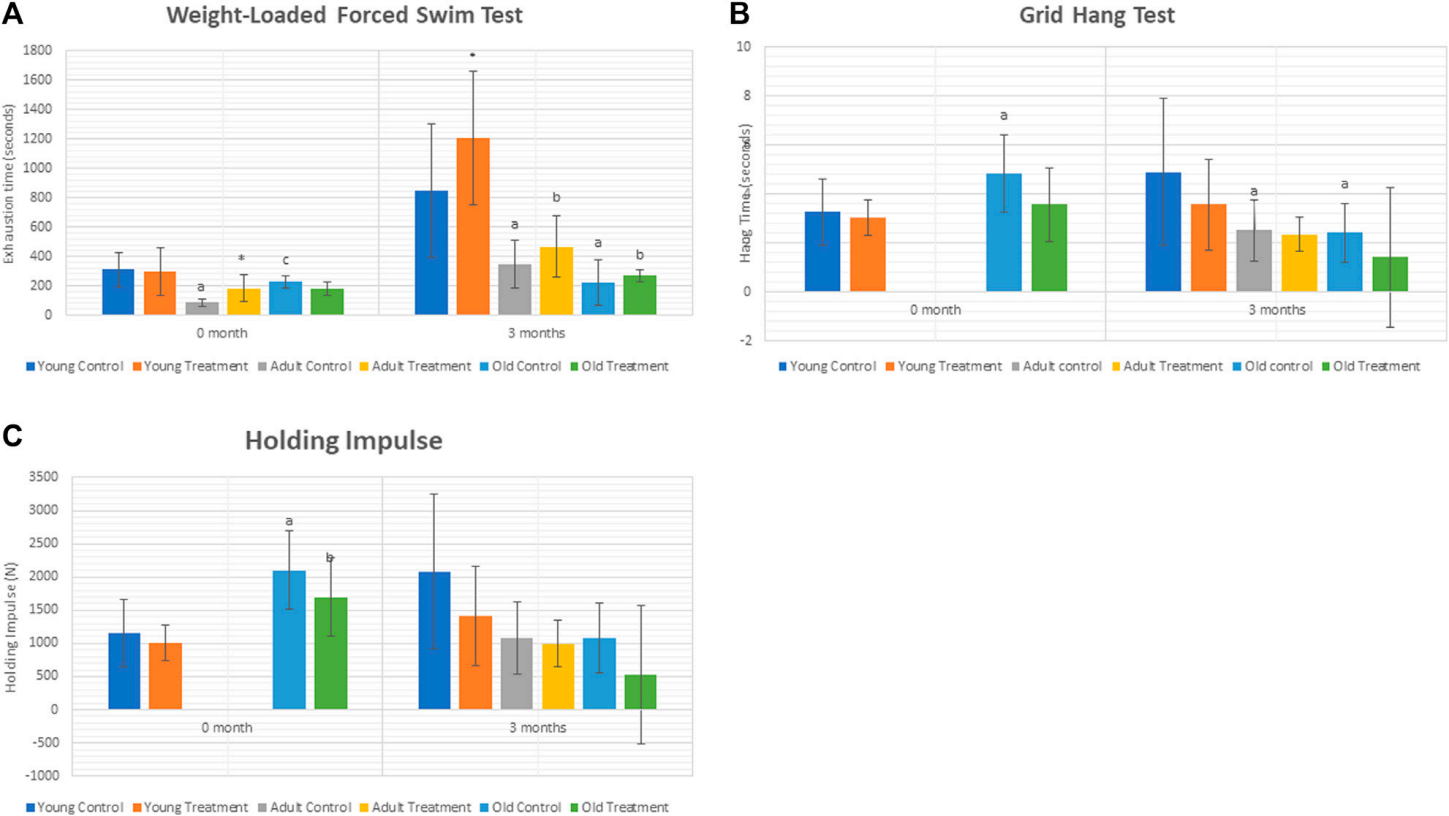
**(A)** Weight-loaded performance swim test. **(B)**. Hang time. **(C)** Holding Impulse. Data are presented as mean ± SD. *p < 0.05, significantly different compared to control, ^a^ *p* < 0.05, significantly different compared to young control rats; ^b^p < 0.05, significantly different compared to young treated rats; ^c^p < 0.05, significantly different compared to adult control rats. Data was analysed using one way ANOVA with a post hoc LSD.

The exhaustion time was significantly decreased at 3 months in adult control rats (AC) and old control rats (OC) as compared to young control rats (YC) **(** Figure 1A **).** Treatment with TRF significantly increased the exhaustion time at 3 months of young treated rats (YT) as compared to YC. No significant change was observed in the adult and old treated groups after 3 months of TRF treatment.

As for the grid hang test, we did not obtain any significant differences between the control and treated groups of rats.

### 3.2 Measurement of hang time and holding impulse

Results showed a significant decrease in the hang time of adult and old control rats compared to young control rats after 3 months. However, no significant change was observed in the hang time and holding impulse after 3 months of TRF treatment **(** Figure 1B,C **)**.

### 3.3 Differential metabolomic analysis between control and treated groups

Principle component analysis (PCA) of young control rats (YC) against adult control rats (AC) generated a 39.6% variation in AC when compared to YC in the positive mode, whereby the PC1 score was 24.5%, and PC2 score was 15.1% **(** Supplementary Figure S1A **)**. The PCA score plot for negative mode described a variation of 42.3% with 27.1% of PC1 and 15.2% of PC2 **(** Supplementary Figure S1B **)**. PCA score plot showed good separation in positive mode but slight overlapping in negative mode.

PCA of YC against old control rats (OC) generated 52.4% variation in OC compared to YC in the positive mode whereby the PC1 score was 39% and PC2 score was13.4% **(** Supplementary Figure S1C **)**. The PCA score plot for negative mode described a variation of 56.2% with 42.9% of PC1 and 13.3% of PC2 (Supplementary Figure S1D). PCA score plot showed good separation in both positive and negative modes.

PCA of AC against OC generated 53.3% variation in OC compared to AC in the positive mode, whereby the PC1 score was 38.7%, and the PC2 score was 14.6% (Supplementary Figure S1E). The PCA score plot for negative mode described a variation of 59.8% with 45.4% of PC1 and 14.4% of PC2 (Supplementary Figure S1F). PCA score plot showed good separation in both positive and negative modes.

PCA of YC against young treated (YT) generated a 35.7% variation in YT compared to YC in the positive mode, whereby the PC1 score was 20.7%, and the PC2 score was 15% **(** Supplementary Figure 2A **).** The PCA score plot for negative mode described a variation of 43.7% with 24.8% of PC1 and 18.9% of PC2 **(** Supplementary Figure 2B). PCA score plot showed overlapping in both positive and negative modes.

PCA of AC against adult treated (AT) showed 39.1% variation in AT compared to AC in the positive mode whereby PCI score was 22.3%, and PC2 score was 16.8% (Supplementary Figure 2C). The PCA score plot for negative mode described a variation of 45.3% with 27.5% of PC1 and 17.8% of PC2 (Supplementary Figure 2D). PCA score plot showed overlapping in both positive and negative modes.

PCA of OC against old treated (OT) showed 54% variation in OT compared to OC in the positive mode whereby PC1 score was 40.8%, and PC2 score was 13.2% (Supplementary Figure 2E). The PCA score plot for negative mode described a variation of 54.9% with 35.3% of PC1 and 19.6% of PC2 (Supplementary Figure 2F). PCA score plot showed overlapping in both positive and negative modes.

### 3.4 Metabolites profiling and regulation in control and treated groups of rats

Control groups were compared between young, adult and old groups to study the effect of age on the expression of metabolites. A comparison between YC and AC generated 19 identified metabolites from 81 metabolic features (Table 1). L-Palmitoyl carnitine was the most up-regulated metabolite in AC, followed by decanoyl carnitine and glycerol 3-phosphate. In contrast, 3-phosphoglyceric acid was the most down-regulated metabolite followed by several other metabolites such as phosphoenol pyruvic acid, aspartyl phenylalanine and nicotinamide adenine dinucleotide. A comparison between YC and OC generated a total of 53 identified metabolites from 81 metabolic features (Table 2). Decanoyl carnitine was the most up-regulated metabolite in OC followed by L-palmitoyl carnitine. In contrast, the majority of the metabolites in OC were downregulated such as aspartyl phenylalanine (the most down-regulated metabolite) followed by amino acids such as arginine, aspartic acid, glutamic acid, histidine, leucine/isoleucine, lysine, phenylalanine, tryptophan, tyrosine and valine. A comparison between AC and OC generated 53 identified metabolites from 81 metabolic features (Table 3). Glycerol 3-phosphate was the most up-regulated metabolite in OC followed by mannose 6-phosphate/fructose 1-phosphate/fructose 6-phosphate/glucose 6-phosphate and 3-phosphoglyceric acid. In contrast, most of the metabolites in OC were downregulated with leucyl-glycine being the most down-regulated metabolite followed by other amino acids.

**TABLE 1:** List of significant metabolites in AC against YC.

Mode	Metabolite	ID	MW	RT	FC
+	2,4-Dihydroxybenzophenone	mzc604	214.0623	0.547	+1.19
+	Aspartylphenylalanine	HMDB0000706	280.1055	3.793	−12.88
+	Bis(4-ethylbenzylidene)sorbitol	mzc7437	414.2036	10.691	−1.26
+	Cytosine	HMDB0000630	111.0434	0.722	+1.40
+	Decanoylcarnitine	HMDB0000651	315.2402	8.324	+4.69
+	Ketamine	HMDB0015352	237.0916	4.973	−2.12
+	L-Palmitoylcarnitine	HMDB0000222	399.3341	11.574	+10.74
+	Methacholine	HMDB0015654	159.1257	0.874	−3.12
+	Nicotinamide adenine dinucleotide	HMDB0000902	663.108	1.151	−7.19
+	Pipecolic acid	HMDB0000070/HMDB0005960/HMDB0000716	129.079	0.821	−1.70
+	Spermine	HMDB0001256	202.2156	0.369	−2.26
+	Uric acid	HMDB0000289	168.0282	0.955	+2.97 (+2.90)
+	Xylazine	HMDB0259938	220.1031	5.268	−3.05
−	3-Phosphoglyceric acid	HMDB0000807	185.9922	0.524	−40.86
−	Citric acid	HMDB0000094	192.0262	0.911	+4.19
−	Glyceric acid	HMDB0000139	106.0256	0.596	−2.41
−	Glycerol 3-phosphate	HMDB0000126	172.0128	0.522	+7.30
−	Mannose 6-phosphate/Fructose 1-phosphate/Beta-D-Fructose 6-phosphate/Glucose 1-phosphate/	HMDB0001078/HMDB0001076/HMDB0003971/HMDB0001586/HMDB0001401	260.0295	0.517	−2.98
−	Phosphoenolpyruvic acid	HMDB0000263	167.9816	0.601	−18.07

YC, as denominator.

FC: positive mode (negative mode).

Potential metabolite identified by cross referencing with mzCloud data (^).

Symbol: (+) increase; (-) decrease.

Acronym: FC (fold change); m/w (molecular weight); RT (retention time).

**TABLE 2:** List of metabolites in OC against YC.

Mode	Metabolite	ID	MW	RT	FC
+	2,6-Dimethylpyrazine/2,5-Dimethylpyrazine	HMDB0035248/HMDB0035289	108.06878	0.494	−1.40
+	2-Amino-4-methylpyrimidine	mzc3275	109.06411	0.488	−1.66
+	2-Hydroxycinnamic acid/4-Hydroxycinnamic acid	HMDB0002641/HMDB0002035	164.04717	1.255	−2.27
+	3-Methylhistidine	HMDB0000479	169.08486	0.494	−1.40
+	4-Hydroxybenzaldehyde	HMDB0011718	122.03683	1.255	−2.19
+	Arginine	HMDB0003416/HMDB0000517	174.11149	0.486	−2.86
+	Aspartic acid	HMDB0000191/HMDB0006483	133.0374	0.503	−3.48 (−5.12)
+	Aspartylphenylalanine	HMDB0000706	280.1055	3.793	−20.77
+	Bis(4-ethylbenzylidene)sorbitol	mzc7437	414.20355	10.691	−1.39
+	Carnosine	HMDB0000033	226.1062	0.485	−1.30
+	Citicoline	HMDB0001413	488.10662	0.627	−1.97
+	Creatine	HMDB0000064	131.06931	0.565	−1.11
+	Creatinine	HMDB0000562	113.05898	0.537	−1.41
+	Decanoyl carnitine	HMDB0000651	315.2402	8.324	+25.93
+	Glutamic acid/Glutamic acid	HMDB0000148/HMDB0003339	147.05284	0.517	−2.08
+	Histidine	HMDB0000177	155.06926	0.487	−1.74 (−1.98)
+	Hypoxanthine	HMDB0000157	136.03832	1.061	−1.92 (−2.27)
+	Indoleacrylic acid	HMDB0000734	187.06315	3.727	−2.27
+	Inosine	HMDB0000195	268.08026	2.32	−1.42
+	Ketamine	HMDB0015352	237.09164	4.973	−13.98
+	Leucine/Isoleucine/Norleucine	HMDB0013773/HMDB0000687/HMDB0000172/HMDB0001645	131.09451	1.393	−3.08
+	Leucyl-Glycine	HMDB0028929	188.11587	3.148	−5.71
+	L-Palmitoylcarnitine	HMDB0000222	399.33412	11.574	+13.87
+	Lysine	HMDB0003405/HMDB0000182	146.10531	0.425	−1.99
+	Methacholine	HMDB0015654	159.12569	0.874	−2.32
+	Methionine	HMDB0000696	149.05078	0.833	−3.34
+	N6-Me-Adenosine	mzc10245	281.11165	1.018	−2.16
+	Phenylalanine	HMDB0000159/METPA0264	165.07877	2.956	−2.54 (-2.99)
+	Prolylglycine	HMDB0011178	172.08458	0.631	−3.07
+	Prolylleucine	mzc559	228.14719	1.275	+2.80
+	Pyroglutamic acid	HMDB0000267	129.04241	1.087	+1.31
+	Spermine	HMDB0001256	202.21555	0.369	−2.11
+	Tryptophan	HMDB0013609/HMDB0000929	204.08963	3.732	−2.27 (−2.40)
+	Tyrosine	HMDB0000158/HMDB0006050	181.07365	1.17	−2.28 (−2.43)
+	Valine	HMDB0000883	117.07904	0.732	−2.18
+	Valyl-Proline	HMDB0029135	214.13124	3.09	−5.20
+	Xylazine	HMDB0259938	220.10305	5.268	+3.20
−	1-Methylhistidine/3-Methylhistidine/α-Methyl-DL-histidine	HMDB0000001/HMDB0000479/mzc792	169.08425	0.486	−1.40
−	3-Phosphoglyceric acid	HMDB0000807	185.99215	0.524	−3.69
−	Anserine	HMDB0000194	240.12177	0.487	−1.31
−	Arginine	HMDB0003416/HMDB0000517	174.11074	0.471	−2.78
−	Citric acid	HMDB0000094	192.02623	0.911	+2.83
−	Glutathione	HMDB0062697	307.08357	0.88	−3.11
−	Glyceric acid	HMDB0000139	106.02556	0.596	−8.24
−	Glycerol 3-phosphate	HMDB0000126	172.01282	0.522	+4.85
−	Malic acid	HMDB0031518/HMDB0000156	134.02045	0.668	−1.38
−	Mannose 6-phosphate/Fructose 1-phosphate/Beta-D-Fructose 6-phosphate/Glucose 1-phosphate/	HMDB0001078/HMDB0001076/HMDB0003971/HMDB0001586/HMDB0001401	260.02945	0.517	+3.83
−	N-Acetylneuraminic acid	HMDB0000230	309.10575	0.587	−2.35
−	Pantothenic acid	HMDB0000210	219.11018	3.474	−1.98
−	Ribose 1-phosphate/Ribose 5-phosphate/Xylulose 5-phosphate/Ribulose 5-phosphate	HMDB0001489/HMDB0001548/HMDB0000868/METPA0382/HMDB0000618/METPA0129	230.01865	0.53	−1.37
−	S-Lactoylglutathione	HMDB0001066	379.10454	1.988	−3.39
−	Taurine	HMDB0000251	125.01358	0.504	−1.13
−	Uridine	HMDB0000296	244.06927	1.374	−1.69
−	Xanthine	HMDB0000292	152.03248	1.262	−2.88
−	Xanthosine	HMDB0000299	284.07556	3.058	−2.82

YC, as denominator.

FC: positive mode (negative mode).

Potential metabolite identified by cross referencing with mzCloud data (^).

Symbol: (+) increase; (-) decrease.

Acronym: FC (fold change); m/w (molecular weight); RT (retention time).

**TABLE 3:** List of metabolites in AC vs. OC.

Mode	Metabolite	ID	MW	RT	FC
+	2,4-Dihydroxybenzophenone	mzc604	0.547	75	−1.44
+	2,6-Dimethylpyrazine/2,5-Dimethylpyrazine	HMDB0035248/HMDB0035289	0.494	73.8	−1.46
+	2-Amino-4-methylpyrimidine	mzc3275	0.488	80.6	−1.63
+	2-Hydroxycinnamic acid/4-Hydroxycinnamic acid	HMDB0002641/HMDB0002035	1.255	87.9	−2.39
+	3-Methylhistidine	HMDB0000479	0.494	95.9	−1.46 (−1.63)
+	4-Hydroxybenzaldehyde	HMDB0011718	1.255	70.3	−2.30
+	Acetylcholine	HMDB0000895	0.655	89.5	−1.27
+	Arginine	HMDB0003416/HMDB0000517	0.486	94	−2.72 (−2.60)
+	Aspartic acid	HMDB0000191/HMDB0006483	0.503	87.7	−3.96 (−3.52)
+	Carnosine	HMDB0000033	0.485	92.9	−1.24
+	Citicoline	HMDB0001413	0.627	85.2	−1.48
+	Creatine	HMDB0000064	0.565	96.1	−1.17
+	Creatinine	HMDB0000562	0.537	93.1	−1.28
+	Cytidine	HMDB0000089	0.725	88.8	−2.05
+	Cytosine	HMDB0000630	0.722	93	−2.44
+	Decanoylcarnitine	HMDB0000651	8.324	83	+5.53
+	Glutamic acid/Glutamic acid	HMDB0000148/HMDB0003339	0.517	94.8	−1.90
+	Histidine	HMDB0000177	0.487	94.9	−1.62 (−1.92)
+	Hypoxanthine	HMDB0000157	1.061	95.5	−2.44 (−2.96)
+	Indoleacrylic acid	HMDB0000734	3.727	92.6	−2.51
+	Inosine	HMDB0000195	2.32	72	−1.69
+	Leucine/Isoleucine/Norleucine	HMDB0013773/HMDB0000687/HMDB0000172/HMDB0001645	1.393	94.6	−3.41
+	Leucyl-Glycine	HMDB0028929	3.148	91.7	−4.65
+	Lysine	HMDB0003405/HMDB0000182	0.425	94.2	−2.58
+	Methionine	HMDB0000696	0.833	89	−2.82
+	N6-Me-Adenosine	mzc10245	1.018	71.7	−1.88
+	N-Acetylornithine	HMDB0003357	0.635	76	−1.30
+	Nicotinamide adenine dinucleotide	HMDB0000902	1.151	91.2	+12.81
+	Phenylalanine	HMDB0000159/METPA0264	2.956	95.3	−3.00 (−3.02)
+	Prolylleucine	mzc559	1.275	85.5	+3.17
+	Pyroglutamic acid	HMDB0000267	1.087	82.2	+1.48
+	Tryptophan	HMDB0013609/HMDB0000929	3.732	95.5	−2.51 (−2.62)
+	Tyrosine	HMDB0000158/HMDB0006050	1.17	87.3	−2.33 (−2.29)
+	Uric acid	HMDB0000289	0.955	87	−3.26 (−3.53)
+	Valine	HMDB0000883	0.732	90	−1.87
+	Valyl-Proline	HMDB0029135	3.09	96.1	−4.18
+	Xylazine	HMDB0259938	5.268	93.6	+9.76
−	3-Phosphoglyceric acid	HMDB0000807	185.99215	0.524	+11.06
−	Anserine	HMDB0000194	240.12177	0.487	−1.30
−	Glutamine	HMDB0003423/HMDB0000641	146.06811	0.499	+1.70
−	Glutathione	HMDB0062697	307.08357	0.88	−4.21
−	Glyceric acid	HMDB0000139	106.02556	0.596	−3.42
−	Glycerol 3-phosphate	HMDB0000126	172.01282	0.522	+35.38
−	Malic acid	HMDB0031518/HMDB0000156	134.02045	0.668	−1.47
−	Mannose 6-phosphate/Fructose 1-phosphate/Beta-D-Fructose 6-phosphate/Glucose 1-phosphate/	HMDB0001078/HMDB0001076/HMDB0003971/HMDB0001586/HMDB0001401	260.02945	0.517	+11.41
−	N-Acetylneuraminic acid	HMDB0000230	309.10575	0.587	−2.13
−	Pantothenic acid	HMDB0000210	219.11018	3.474	−1.90
−	Ribose 1-phosphate/Ribose 5-phosphate/Xylulose 5-phosphate/Ribulose 5-phosphate	HMDB0001489/HMDB0001548/HMDB0000868/METPA0382/HMDB0000618/METPA0129	230.01865	0.53	−1.65
−	S-Lactoylglutathione	HMDB0001066	379.10454	1.988	−2.06
−	Taurine	HMDB0000251	125.01358	0.504	−1.19
−	Uridine	HMDB0000296	244.06927	1.374	−1.66
−	Xanthine	HMDB0000292	152.03248	1.262	−4.02
−	Xanthosine	HMDB0000299	284.07556	3.058	−3.18

AC, as denominator.

FC: positive mode (negative mode).

Potential metabolite identified by cross referencing with mzCloud data (^).

Symbol: (+) increase; (-) decrease.

Acronym: FC (fold change); m/w (molecular weight); RT (retention time).

Profiled metabolites identified in the control groups were compared based on fold-change. Based on these comparisons, carnitine was the major metabolite group involved in all three control groups. Decanoyl carnitine was found to be up-regulated by 4.69-fold in AC against YC (Table 1), 25.93-fold in OC against YC (Table 2) and 5.53-fold in OC against AC (Table 3). Besides that, L-palmitoyl carnitine was up-regulated by 10.74-fold in AC against YC (Table 1) and 13.87-fold in OC against YC (Table 2). Glycerol 3-phosphate was another major metabolite that was found to be up-regulated in all three control groups, with an increase of 7.30-fold in AC against YC (Table 1) and 4.85-fold in OC against YC (Table 2) and 35.38-fold in OC against AC (Table 3). Mannose 6-phosphate/Fructose 1-phosphate/beta-D-Fructose-6-phosphate/Glucose 1-phosphate was found to be up-regulated in both OC against YC **(** Table 2) (3.83-fold) and OC against AC (Table 3) (11.41-fold).

In contrast, phosphoenolpyruvate (PEP) metabolite showed an 18.07-fold decrease in AC against YC (Table 1). Another major metabolite group that was found to be involved in all three groups is glycerate, which includes glyceric acid and 3-phosphoglyceric acid. Glyceric acid was found to be down-regulated by 2.41-fold in AC against YC (Table 1), 8.24-fold in OC against YC (Table 2) and 3.42-fold in OC against AC (Table 3). 3-Phosphoglyceric acid was down-regulated by 40.86-fold in AC against YC (Table 1) and 3.69-fold in OC against YC (Table 2). However, it was up-regulated by 11.06-fold in OC against AC (Table 3).

Treatment groups were compared based on fold change against untreated control groups of young, adult and old rats. A total of 81 metabolic features were identified. Comparisons between YC and YT groups identified seven significant metabolites (Table 4). Decanoyl carnitine and L-palmitoyl carnitine showed an up-regulation of 2.81-fold and 7.41-fold, respectively in TRF-treated rats. Aspartyl phenylalanine and nicotinamide adenine dinucleotide (NAD^+^) were down-regulated by 3.37-fold and 2.27-fold, respectively. A comparison between AC and AT groups identified 16 significant metabolites (Table 5). NAD + showed the highest up-regulation (7.79-fold) while citric acid showed the most down-regulation (17.30-fold). Spermine was up-regulated by 3.56-fold and aspartic acid by 1.26-fold. A comparison between OC and OT groups identified 11 significant metabolites (Table 6). Nine of the 11 metabolites showed up-regulation, with aspartic acid being the most up-regulated by 8.98-fold, followed by N6-Me-Adenosine (4.54-fold), phenylalanine (2.13-fold), tryptophan (2.07-fold) and histidine (1.84-fold). Only carnitine and L-acetyl carnitine showed down-regulation by 1.15-fold and 1.19-fold, respectively.

**TABLE 4:** List of metabolites in YT against YC.

Mode	Metabolite	ID	MW	RT	FC
+	Aspartylphenylalanine	HMDB0000706	280.1055	3.793	−3.37
+	Decanoylcarnitine	HMDB0000651	315.2402	8.324	+2.81
+	L-Palmitoylcarnitine	HMDB0000222	399.33412	11.574	+7.41
+	Methacholine	HMDB0015654	159.12569	0.874	−1.95
+	Nicotinamide adenine dinucleotide	HMDB0000902	663.10799	1.151	−2.27
+	Prolylleucine	mzc559	228.14719	1.275	−1.52
−	Xanthosine	HMDB0000299	284.07556	3.058	−1.87

YC, as denominator.

FC: positive mode (negative mode).

Potential metabolite identified by cross referencing with mzCloud data (^).

Symbol: (+) increase; (-) decrease.

Acronym: FC (fold change); m/w (molecular weight); RT (retention time).

**TABLE 5:** List of metabolites in AT against AC.

Mode	Metabolite	ID	MW	RT	FC
+	Acetylcholine	HMDB0000895	145.11005	0.655	−1.21
+	Adenine	HMDB0000034	135.05422	0.842	+2.05
+	Adenosine monophosphate/3′-Adenosine monophosphate	HMDB0000045/HMDB0003540	347.06241	0.857	+1.54
+	Aspartic acid	HMDB0000191/HMDB0006483	133.0374	0.503	+1.26
+	Ketamine	HMDB0015352	237.09164	4.973	+4.68
+	N6-Me-Adenosine	mzc10245	281.11165	1.018	+1.61
+	N-Acetylaspartylglutamic acid	HMDB0001067	304.08996	1.421	+1.33
+	Nicotinamide adenine dinucleotide	HMDB0000902	663.10799	1.151	+7.79
+	Prolylglycine	HMDB0011178	172.08458	0.631	+1.95
+	Prolylleucine	mzc559	228.14719	1.275	+1.54
+	Pyroglutamic acid	HMDB0000267	129.04241	1.087	+1.40
+	Spermine	HMDB0001256	202.21555	0.369	+3.56
+	Uric acid	HMDB0000289	168.02815	0.955	−1.54
−	Citric acid	HMDB0000094	192.02623	0.911	−17.30
−	Glycerol 3-phosphate	HMDB0000126	172.01282	0.522	+3.74
−	Mannose 6-phosphate/Fructose 1-phosphate/Beta-D-Fructose 6-phosphate/Glucose 1-phosphate/	HMDB0001078/HMDB0001076/HMDB0003971/HMDB0001586/HMDB0001401	260.02945	0.517	−2.84

AC, as denominator.

FC: positive mode (negative mode).

Potential metabolite identified by cross referencing with mzCloud data (^).

Symbol: (+) increase; (-) decrease.

Acronym: FC (fold change); m/w (molecular weight); RT (retention time).

**TABLE 6:** List of metabolites in OT against OC.

Mode	Metabolite	ID	MW	RT	FC
+	Carnitine	HMDB0000062	161.10487	0.547	−1.15
+	Cytidine	HMDB0000089	243.08509	0.725	+2.80
+	Indoleacrylic acid	HMDB0000734	187.06315	3.727	+2.07
+	L-Acetylcarnitine	HMDB0000201	203.11546	0.909	−1.19
+	N6-Me-Adenosine	mzc10245	281.11165	1.018	+4.54
+	Phenylalanine	HMDB0000159/METPA0264	165.07877	2.956	+2.13
+	Tryptophan	HMDB0013609/HMDB0000929	204.08963	3.732	+2.07
−	Aspartic acid	HMDB0000191/HMDB0006483	133.03642	0.498	+8.98
−	Histidine	HMDB0000177	155.06853	0.479	+1.84
−	N-Acetylneuraminic acid	HMDB0000230	309.10575	0.587	+1.36
−	Xanthine	HMDB0000292	152.03248	1.262	+2.43

OC, as denominator.

FC: positive mode (negative mode).

Potential metabolite identified by cross referencing with mzCloud data (^).

Symbol: (+) increase; (-) decrease.

Acronym: FC (fold change); m/w (molecular weight); RT (retention time).

### 3.5 Biochemical pathways analysis of muscle metabolomes in control and treated groups of rats

**FIGURE 2:**
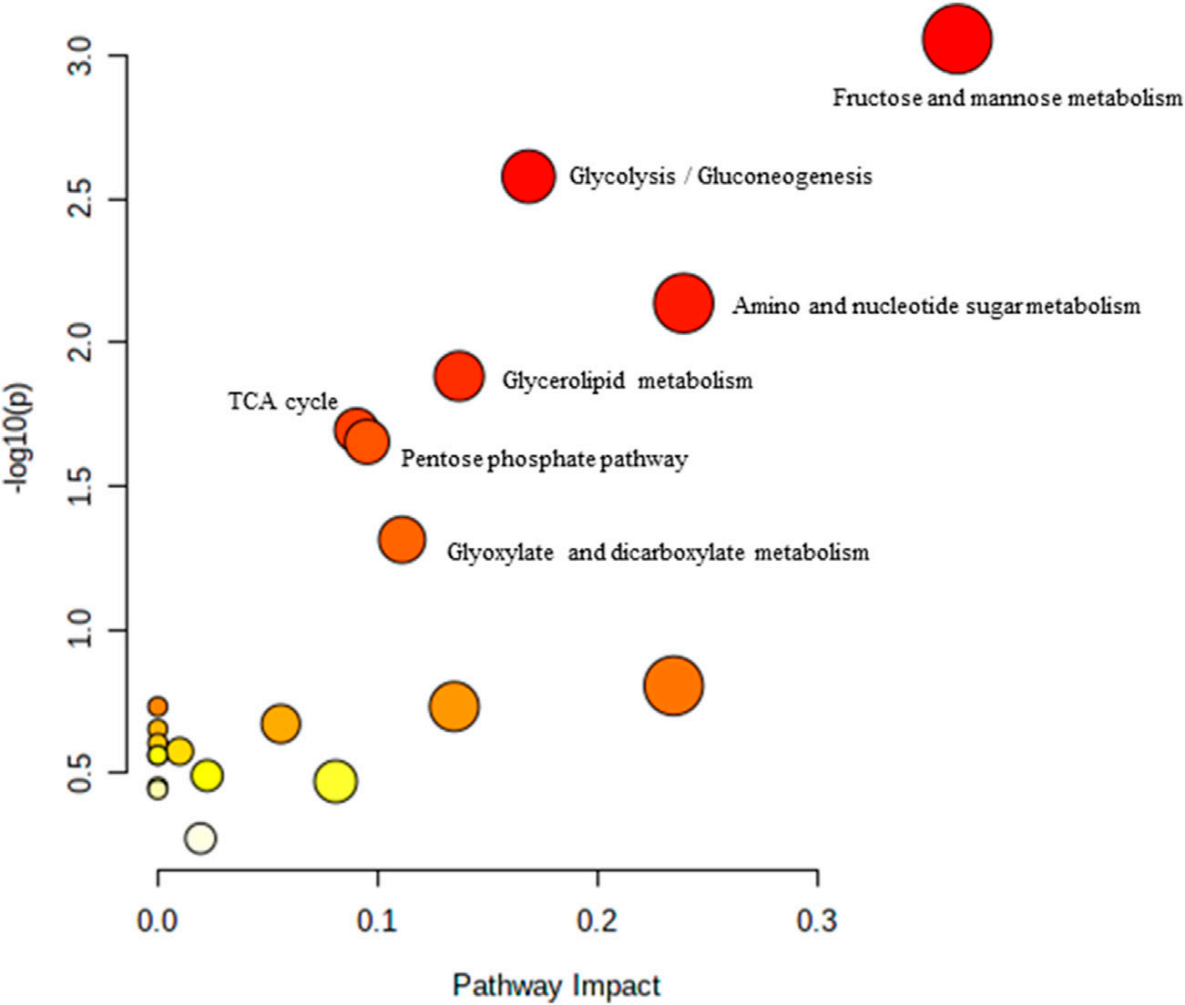
Biochemical pathway analysis of metabolites profiled for YC vs. AC.

**FIGURE 3:**
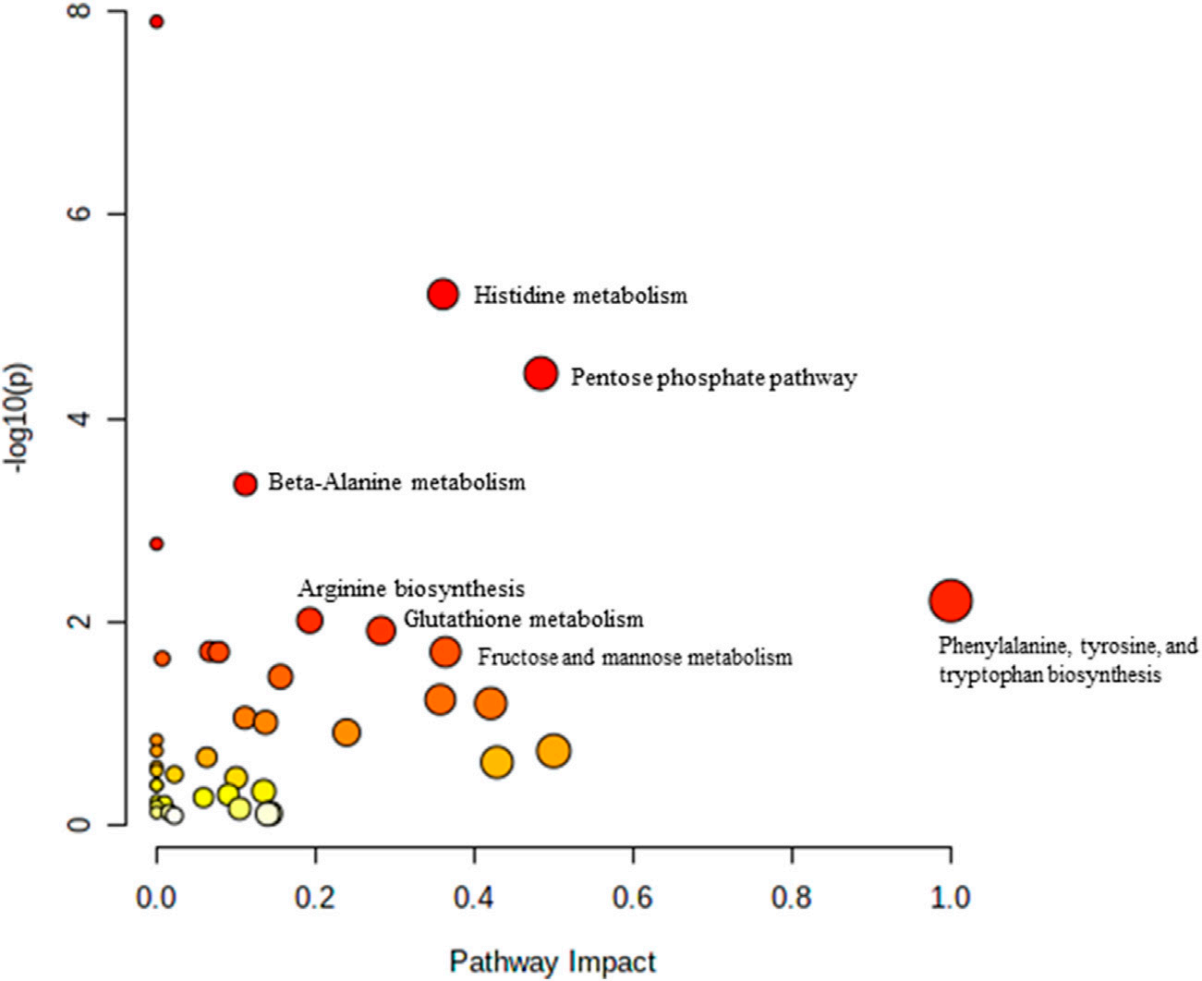
Biochemical pathway analysis of metabolites profiled for YC vs. OC.

**FIGURE 4:**
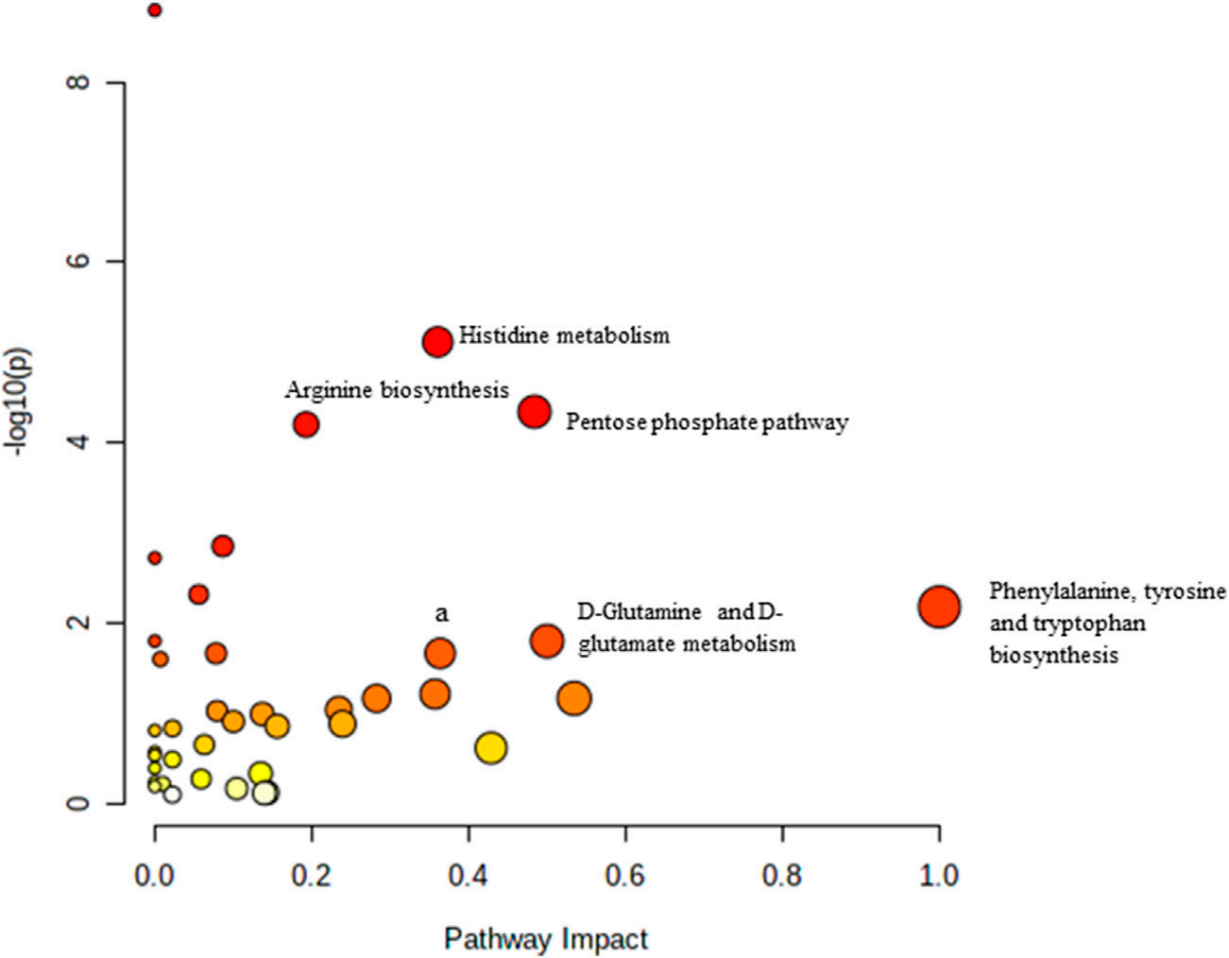
Biochemical pathway analysis of metabolites profiled for AC vs. OC. a: Fructose and mannose metabolism.

Pathway analysis was performed using MetaboAnalyst on the muscle metabolomes expression during ageing. A comparison of profiled metabolites between AC and YC generated 21 biochemical pathways (Figure 2). The significant pathways involved were primarily fructose and mannose metabolism, glycolysis/gluconeogenesis, amino sugar and nucleotide sugar metabolism, glycerolipid metabolism, TCA cycle, pentose phosphate pathway and glyoxylate and dicarboxylate metabolism **(** Supplementary Table S1 **)**. Pathways were identified as significant with a *p*-value <0.05 and an impactor of >1.0. Metabolites profiled in OC compared to YC generated 39 biochemical pathways (Figure 3). The significant pathways involved were primarily histidine metabolism; pentose phosphate pathway; beta-alanine metabolism; phenylalanine, tyrosine and tryptophan biosynthesis; arginine biosynthesis; glutathione metabolism; purine metabolism; pentose and glucuronate interconversions; fructose and mannose metabolism; and arginine and proline metabolism (Supplementary Table S2). Comparison of profiled metabolites in OC and AC generated 39 biochemical pathways (Figure 4). The significant pathways involved were primarily histidine metabolism, pentose phosphate pathway; arginine biosynthesis; phenylalanine, tyrosine and tryptophan biosynthesis; D-glutamine and D-glutamate metabolism and fructose and mannose metabolism (Supplementary Table S3).

**FIGURE 5:**
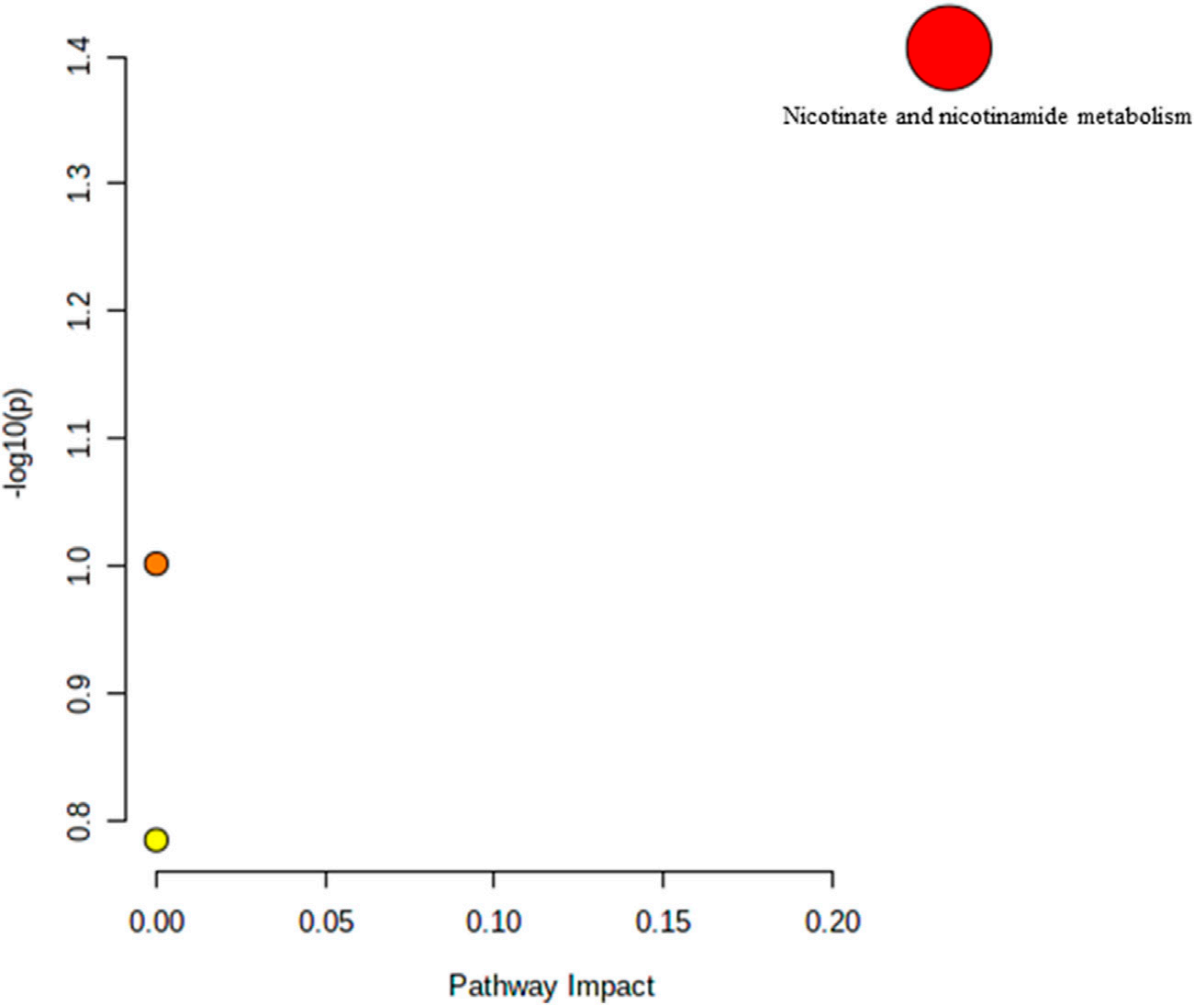
Biochemical pathway analysis of metabolites profiled for YC vs. YT.

**FIGURE 6:**
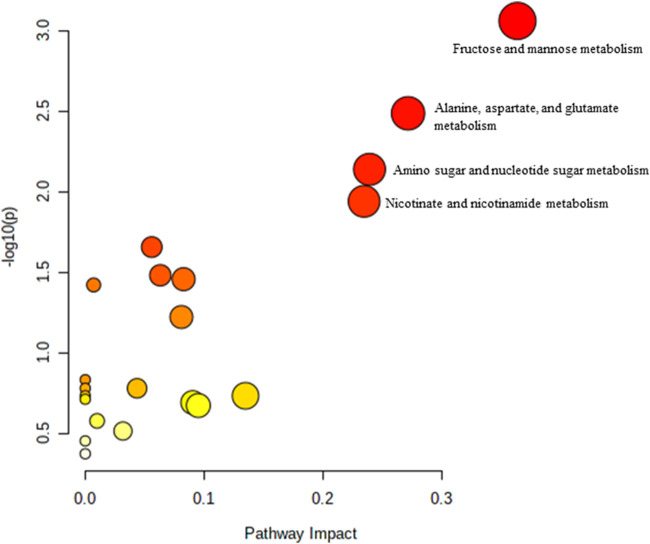
Biochemical pathway analysis of metabolites profiled for AC vs. AT.

**FIGURE 7:**
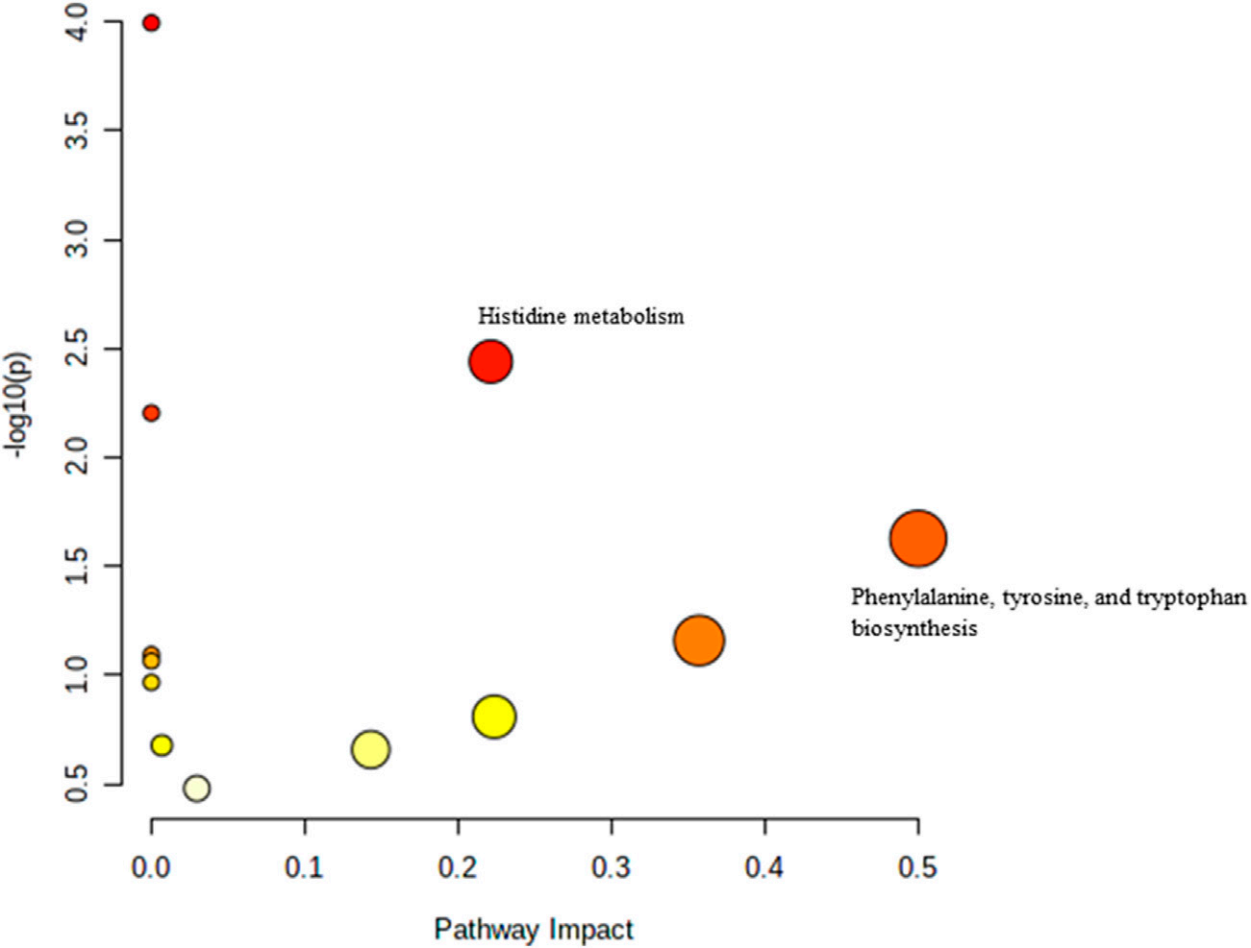
Biochemical pathway analysis of metabolites profiled for OC vs. OT.

The effect of TRF treatment on the metabolic pathway was determined by comparing the muscle metabolomes expression in the TRF-treated group with the untreated control group. A comparison of the profiled metabolites in the TRF-treated group against the control group of the young rats generated three metabolites (Figure 5) whereby nicotinate and nicotinamide metabolism was the significant biochemical pathway **(** Supplementary Table S4). In the adult group, a comparison between the profiled metabolites of AT against AC, generated a total of 21 biochemical pathways (Figure 6), with the significant pathways involved primarily being fructose and mannose metabolism; alanine, aspartate and glutamate metabolism; amino sugar and nucleotide sugar metabolism; and nicotinate and nicotinamide metabolism (Supplementary Table S5). In the old group, a comparison between profiled metabolites of OT against OC, generated 12 biochemical pathways (Figure 7). The significant pathways involved were histidine metabolism and phenylalanine, tyrosine and tryptophan biosynthesis **(** Supplementary Table S6).

## 4 Discussion

Sustaining the health of one’s muscles requires a sufficient intake of vitamin E. In a previous study using animal models, severe tocopherol deficiency has been observed to impair muscle performance even though vitamin E deficiency is rarely reported in humans (Nier et al., 2006; Rafique et al., 2004). Decrease in muscle mass and function in sarcopenia has been reported to be correlated with low vitamin E levels, as shown by the physical performance test. Invecchiare in Chianti study reported that the level of vitamin E daily intake positively correlates with knee extension strength and total the physical performance (Cesari et al., 2004) indicating the requirement of vitamin E in maintaining muscle strength and function.

In this study, physical performance was observed by conducting the weight-loaded performance swim test and the grid hang test. The results of our study showed that muscle strength was reduced with ageing. Treatment with TRF for 3 months however, increased the exhaustion time in weight-loaded performance swim test of young rats eventhough no similar effects were observed in adult and old rats treated with TRF. This might occur because the young TRF-treated rats have more significant muscle regeneration potential than the adult or old TRF-treated rats. The ability of muscles to regenerate relied entirely on the renewal of satellite cells, which eventually declined with age (Dumont et al., 2015). It has been previously reported that ageing causes the number of satellite cells to decrease (Miljkovic et al., 2015). According to a study by Khor et al., 2016, senescent myoblasts have low proliferative capacities, which showed downregulation of myogenic differentiation genes but increased expression of oxidative damage-associated genes. This study may explain the reduced ability of adults and old TRF-treated rats to regenerate muscle in this study.

This study also demonstrated that supplementation of TRF to young, adult and old rats for 3 months could reduce the levels of DNA damage indicating the capacity of TRF to reduce damaged DNA due to excessive ROS generation. This finding was in line with a previous study which reported that tocotrienols are rich vitamin E derivatives that aid in preventing DNA damage brought on by ageing (Chin et al., 2019; Georgousopoulou et al., 2017; Pathak et al., 2021; Taridi et al., 2014). A previous randomised clinical trial involving middle-aged and older adults supplemented with tocotrienol for 6 months showed a significant reduction in DNA damage by 3 months, which is a benefit that persisted through the 6 months of the study (Chin et al., 2019).

In this study, the effect of TRF supplementation on young, adult and old Sprague Dawley rats and its molecular mechanism was elucidated using an untargeted metabolomic approach. The animal model used in this study has been reported to be appropriate for studying sarcopenia (Shavlakadze and Grounds, 2006) (Chai et al., 2011; Schiaffino and Mammucari, 2011; Pötsch et al., 2014; Kob et al., 2015; Tarantini et al., 2019). Aged rat models are the most natural ageing model and have morbidities and mechanisms reasonably similar to those found in humans with sarcopenia (Baek et al., 2020). Rats grow quickly and at around 6 weeks old, they are sexually mature, and after five or 6 months, they are socially mature. An adult animal’s month is roughly similar to 2.5 years in a human (Andreollo et al., 2012). The decline in physical activity that occurs with ageing causes metabolic changes and worsens metabolic abnormalities (Pataky et al., 2021).

**FIGURE 8:**
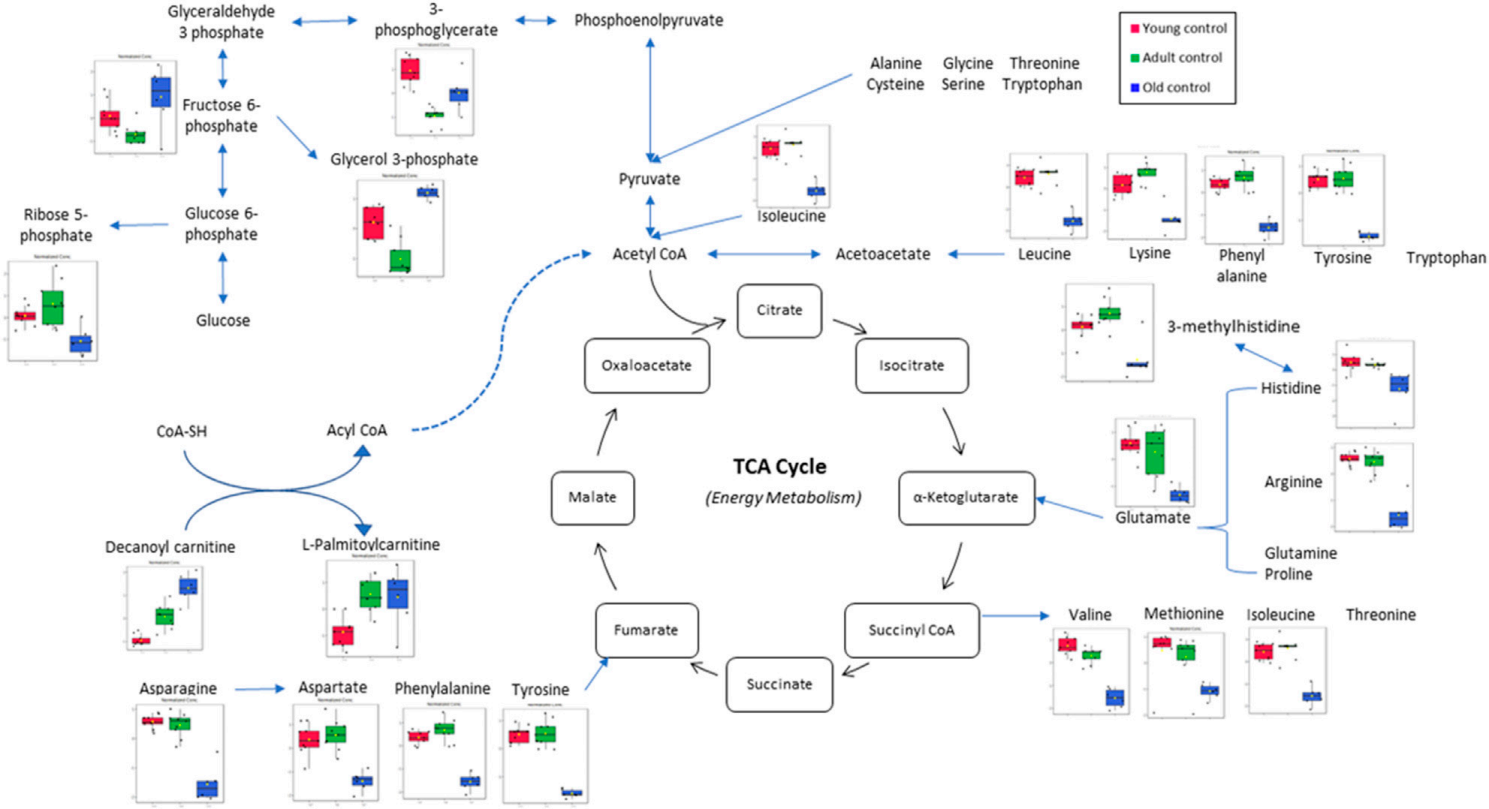
Muscle metabolomes changes in ageing rats. Metabolite concentrations in muscle specimens are depicted graphically and subjected to quantitative analysis. Box and whiskers plots with 95% confidence intervals are presented for quantified amino acids. Analysis was performed using one-way Anova with Tukey’s post hoc analysis. *p < 0.05.

In this study, the metabolites profile of the control groups was compared to provide insight into the metabolites regulated by ageing. Distinctive characteristics of ageing skeletal muscle metabolomes were summarised in Figure 8. Our results showed the reduction of alanine, phenylalanine, tyrosine and glycine as well as the branched-chain amino acids (BCAAs)—isoleucine, leucine and valine in the old control group indicating an imbalance between protein synthesis and breakdown during ageing**.** Interestingly, none of these amino acid alterations was present in the young or adult groups, indicating that ageing affects dysregulated amino acid metabolism. A biomarker of muscle protein degradation, 3-methylhistidine, was noticeably downregulated in the muscle tissue of the elderly control group. This metabolite is not recycled but quantitatively excreted in the urine and has been proposed as an index of muscle protein turnover.

One of the most striking metabolite changes with age is in the level of biochemical markers of glucose metabolism. Levels of glycolytic intermediates decreased significantly in the muscle of adults and old rats. 3-Phosphoglycerate and phosphoenol pyruvate were each detected with decreased fold change in the adult and old control groups besides downregulation of nicotinamide adenine dinucleotide (NAD), a co-enzyme central to metabolism. Glycolysis is the primary pathway of glucose metabolism for energy production (Xiang et al., 2021). A decrease in glycolytic intermediate means that there is an impairment in energy production. It is often believed that ageing causes glucose intolerance, and skeletal muscle is the primary site of glucose uptake (Kohrt and Holloszy, 1995). Previous research reported that resting skeletal muscle glycogen stores were 60% lower in old than young individuals (Meredith et al., 1989). This suggests an age-related impairment in the glycogen synthase pathways (Consitt et al., 2019). Other studies have reported that skeletal muscle glycogen synthase activity is diminished in older adults compared to younger individuals (Pehleman et al., 2005; Poulsen et al., 2005; Biensø et al., 2015).

Carnitine is an amino acid derivative that functions as a transporter of long-chain fatty acids from the cytosol to the mitochondrial matrix for fatty acid β-oxidation (Gnoni et al., 2020). Acylcarnitine is a metabolite of fatty acid transportation whereby fatty acyl CoA will be released from acylcarnitine for fatty acid oxidation. Decanoyl carnitine and L-palmitoyl carnitine are medium-chain acylcarnitine, an intermediate of fatty acid β-oxidation by-products (Ramos-Roman et al., 2012). It is also a marker for incomplete fatty acid oxidation (Mai et al., 2013), and acylcarnitine accumulation is a marker for mitochondrial dysfunction (Al-Bakheit et al., 2016). This study shows an increase in decanoyl carnitine and L-palmitoyl carnitine levels in ageing rats, which is in line with recent reports that suggest the rise of acylcarnitine levels may be caused by the impairment of lipid oxidation or a natural response to an excess lipid supply (Ramos-Roman et al., 2012). It is known that lipid oxidation indeed decreases in elderly individuals (Calles-Escandón and Poehlman, 1997).

Phosphoenolpyruvate is an intermediate for both glycolysis and gluconeogenesis. It can be converted to pyruvate by pyruvate kinase to produce energy (Hamasaki et al., 1978). Recent studies have reported that the ageing of C. elegans is highlighted by a progressive decline in cytosolic phosphoenolpyruvate carboxykinase, which is the enzyme responsible for converting pyruvate to phosphoenolpyruvate in gluconeogenesis (Yuan et al., 2016). This study shows a decrease in phosphoenolpyruvate in the adult control compared to the young control group indicating inefficient glycolysis and gluconeogenesis. Thus, changes in phosphoenolpyruvate with age are likely involved in energy metabolism that acutely affects the physiology of ageing organisms, thus impacting the ageing process (Feng et al., 2016).

In this study, TRF supplemented group was compared with the RBD palm olein supplemented group as a control to provide insight into the different metabolites being regulated by TRF supplementation in young (3 months old), adult (9 months old) and old (21 months old) rats. The RBD palm olein was used in this study as it is the carrier for TRF. It is the liquid form of palm oil that is edible and is commonly used for cooking, which is easily accessible. TRF used in this study was extracted from the Malaysian palm tree *Elaeis guineensis*. Palm-based TRF is easily accessible and has been studied for its therapeutic properties, mainly in Malaysia. On the other hand, the Westerns are more inclined toward annatto oil or rice bran oil-based TRF. What differs between these oils is the amount of vitamin E homologs in them. Annatto and rice bran oil-based TRF encompasses a higher concentration of δ-tocotrienol and a lower amount of tocopherol (Frega et al., 1998; Burdeos et al., 2012; Allen et al., 2017). Contrarily, palm oil is one of the most abundant natural sources of tocotrienols, which contains up to 800 mg/kg weight of the α- and γ-tocotrienol isotypes in crude palm oil (also known as tocotrienol-rich fraction). In palm oil, vitamin E is distributed as 30% tocopherols and 70% tocotrienols (Sen et al., 2010). Therefore, utilising palm-based TRF in this study may produce robust knowledge in the metabolomic analysis as both tocotrienol and tocopherol would affect multiple genes and metabolic pathways (Pang and Chin, 2019).

Rats supplemented with TRF for 3 months showed an increase in spermine in the adult treated group. The same metabolite was downregulated in the adult control rats when compared to young control group. Spermine, which is oxidised by spermine oxidase, is an essential regulator of muscle gene expressions and fibre size. Studies have shown that a repressed level of spermine leads to muscle atrophy (Ceci et al., 2017). The decrease in spermine with ageing observed in this study was counter-regulated by TRF as shown by the increase of spermine with TRF treatment indicating that supplementation of TRF can reverse the repression of spermine due to ageing. Another metabolite that was found to be upregulated with TRF treatment in adult rats was nicotinamide adenine dinucleotide (NAD). Previous research has reported a decline in NAD^+^ concentrations with ageing in a tissue-specific manner (Braidy et al., 2011; Massudi et al., 2012). NAD^+^ is a coenzyme that facilitates redox reactions in all living cells mainly for energy production (Obrador et al., 2021). The upregulation of NAD^+^ in this study after 3 months of TRF supplementation in the adult treated group coincides with the TRF antioxidant properties that may have reversed the ageing-decline of NAD^+^ and promoted energy production.

Another metabolite highly downregulated with TRF treatment in the adult group was citric acid, which shows a 17.30-fold downregulation in the treated group compared to the control group. Citric acid is an intermediate of the tricarboxylic acid (TCA) cycle (Baldwin and Krebs 1981), which is involved in a metabolic pathway converting carbohydrates, proteins and fats into carbon dioxide and water to produce energy. Evidence demonstrates that energy production progressively decreases with age, mainly due to the decline in mitochondrial function (Bratic and Trifunovic 2010). A decrease in the level of citric acid shows usage of the metabolite in the TCA cycle, which in this study is shown in the TRF-treated group. Thus, showing that TRF supplementation reverses the age-associated decrease in energy production.

In this study, glycerol 3-phosphate was highly upregulated with ageing. Glycerol 3-phosphate is produced from the phosphorylation of glycerol by a glycerol kinase. The source of glycerol can be from the breakdown of triacylglycerol or triglycerides *via* lipolysis. Glycerol 3-phosphate is converted to dihydroxyacetone phosphate (DHAP), an intermediate of glycolysis and gluconeogenesis by glycerol 3-phosphate dehydrogenase (mGDPH) utilising NAD^+^ *via* a reversible reaction. Studies have shown that loss of mGDPH attenuates muscle regeneration, while overexpression of it ameliorated dystrophic pathology in mice (Liu et al., 2018). Muscle regeneration decreases with age as ageing causes satellite cells to malfunction, influencing their regenerative and self-renewal capacities (Sousa-Victor et al., 2014). Other studies have also shown how age reduces glycerol 3-phosphate (Nemutlu et al., 2015). The findings of this study showed that there was an accumulation of glycerol 3-phosphate in old rats which was alleviated by TRF treatment. This agrees with a previous study, which reported that TRF increases the proliferation capacity of senescent myoblasts, thus increasing muscle regeneration (Khor et al., 2016). The accumulation of glycerol 3-phosphate was linked with increased NAD^+^, because less NAD^+^ is converted to NADH due to less conversion of glycerol 3-phosphate to DHAP. In this study, an upregulation of NAD^+^ was seen after 3 months of TRF supplementation in the adult group.

As for the muscle metabolomes expression in the old group of rats, several metabolites particularly the amino acids were downregulated. TRF treatment however, increased the expression of aspartic acid, phenylalanine, tryptophan and histidine besides upregulating N6-Me-adenosine, cytidine, xanthine, indoleacrylic acid and N-acetylneuraminic acid in old rats. Aspartic acid is important in glutamate flux through the TCA cycle and glutamate transamination (Birsoy et al., 2015). A previous study showed that aspartic acid levels decreased in old age (Lo et al., 2020) which was similar to the findings of this current study. However, with TRF treatment, the aspartic acid level was upregulated by 8.98-fold in old group of rats. This shows that TRF supplementation reverses the age-declining cause of aspartic acid involvement in biochemical pathways of TCA cycle activity (Lancha et al., 2009).

**FIGURE 9:**
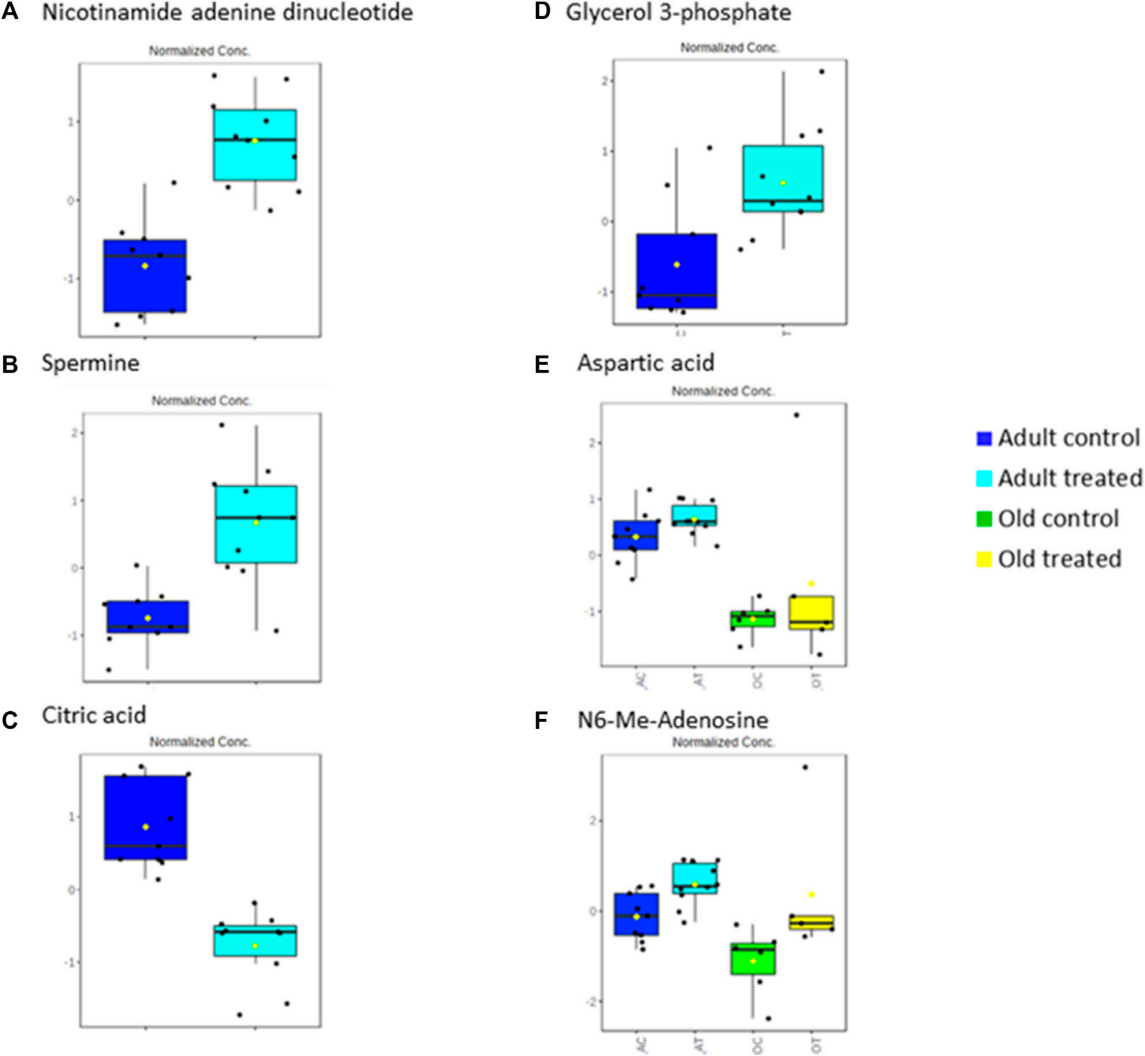
Significant metabolic changes after tocotrienol-rich fraction treatment.

The metabolites profiled also detected an upregulation of N6-Me-Adenosine. Researchers in Bonn, Germany, have reported that adenosine, a metabolite and signaling molecule, can enhance muscle function and brown fat during ageing and obesity. N6-methyladenosine is a methylation product which occurs in the N6-position of adenosine, the most prevalent internal modification of eukaryotic mRNA. Many reports have suggested that N6-methyladenosine modulates gene expression, which regulates cellular processes, including cell self-renewal, differentiation, invasion and apoptosis (He et al., 2019). Recent studies have shown that N6-methyladenosine plays an essential function in skeletal muscle development by regulating myoblast proliferation and differentiation during myogenesis and is the key to biological processes and regulatory pathways during skeletal muscle development (Zhang et al., 2020). N6-methyladenosine can accurately regulate stem cell differentiation and reprogram it during stem cell development by modulating the gene expression involved in the corresponding processes (Wang et al., 2019). Similar to the function of glycerol 3-phosphate, which plays a role in muscle regeneration, our results showed that N6-methyl adenosine was downregulated in old rats when compared to the young and adult control groups. However, after 3 months of TRF supplementation, the concentration of N6-methyl adenosine has increased in both adult and old groups of rats, showing that TRF supplementation might be involved in facilitating the muscle regeneration process. Significant metabolic changes between the control and treated group were summarised in Figure 9.

The muscle performance finding from this study was limited as there were logistic issues due to the Covid-19 pandemic and the lack of proper software to observe the behaviour of the rats during the muscle performance tests. Therefore, further study is needed to explain the effect of TRF on muscle performance. However, the molecular analysis of the mechanism of TRF in reducing the level of oxidative stress in muscle supported by the modulation of the metabolic pathways observed in the present study strengthens the properties of TRF as a potential agent to prevent age-related muscle degeneration and sarcopenia.

## 5 Conclusion

Our data indicate that TRF improves muscular function, and delays muscle degeneration due to ageing in Sprague Dawley rats. Our findings also showed the principal variations in muscle metabolomes due to ageing as shown in the old and young groups of rats which were related to tissue turnover, fibre size and mitochondrial function. The effect of tocotrienol-rich fraction on reducing several metabolites such as glycerol 3-phosphate and upregulating several amino acids and N6-Me-adenosine are likely to ameliorate muscle regeneration and cell self-renewal in the old group of rats. The mechanisms underlying the modulation in skeletal muscle metabolomes expression following TRF supplementation positively impact the potential treatment of sarcopenia in aged rats.

## Data Availability

The raw data supporting the conclusions of this article will be made available by the authors, without undue reservation.

## References

[B1] Al-Bakheit A. Traka 'a. Saha M. Mithen S. Melchini R. Melchini A. (2016). Accumulation of palmitoylcarnitine and its effect on pro-inflammatory pathways and calcium influx in prostate cancer. Prostate 76, 1326–1337. 10.1002/pros.23222 27403764 PMC4996340

[B2] Allen L. Ramalingam L. Menikdiwela K. Scoggin S. Shen C. L. Tomison M. D. (2017). Effects of delta-tocotrienol on obesity-related adipocyte hypertrophy, inflammation and hepatic steatosis in high-fat-fed mice. J. Nutr. Biochem. 48, 128–137. 10.1016/j.jnutbio.2017.07.003 28825992

[B3] Andreollo N. A. Santos E. F. Araújo M. R. Lopes L. R. (2012). Idade dos ratos versus idade humana: Qual é a relação? ABCD, Arq. Bras. Cir. Dig. 25, 49–51. 10.1590/s0102-67202012000100011 22569979

[B4] Baek K-W. Jung Y-K. Kim J-S. Park J. S. Hah Y-S. Kim S-J. (2020). Rodent model of muscular atrophy for sarcopenia study. J. Bone Metab. 27, 97–110. 10.11005/jbm.2020.27.2.97 32572370 PMC7297619

[B5] Baldwin J. E. Krebs H. (1981). The evolution of metabolic cycles. Nature 291, 381–382. 10.1038/291381a0 7242661

[B6] Beard J. R. Officer A. de Carvalho I. A. Sadana R. Pot A. M. Michel J. P. (2016). The world report on ageing and health: A policy framework for healthy ageing. Lancet 387, 2145–2154. 10.1016/s0140-6736(15)00516-4 26520231 PMC4848186

[B7] Bhattacharya S. Granger C. B. Craig D. Haynes C. Bain J. Stevens R. D. (2014). Validation of the association between a branched chain amino acid metabolite profile and extremes of coronary artery disease in patients referred for cardiac catheterization. Atherosclerosis 232, 191–196. 10.1016/j.atherosclerosis.2013.10.036 24401236 PMC4784695

[B8] Biensø R. S. Olesen J. Gliemann L. Schmidt J. F. Matzen M. S. Wojtaszewski J. F. (2015). 'Effects of exercise training on regulation of skeletal muscle glucose metabolism in elderly men. J. Gerontol. A Biol. Sci. Med. Sci. 70, 866–872. 25991826 10.1093/gerona/glv012

[B9] Birsoy K. Wang T. Chen W. W. Freinkman E. Abu-Remaileh M. Sabatini D. M. (2015). An essential role of the mitochondrial electron transport chain in cell proliferation is to enable aspartate synthesis. Cell. 162, 540–551. 10.1016/j.cell.2015.07.016 26232224 PMC4522279

[B10] Braidy N. Guillemin G. J. Mansour H. Chan-Ling T. Poljak A. Grant R. (2011). Age related changes in NAD+ metabolism oxidative stress and Sirt1 activity in wistar rats. PLOS ONE 6, e19194. 10.1371/journal.pone.0019194 21541336 PMC3082551

[B11] Bratic I. Trifunovic A. (2010). Mitochondrial energy metabolism and ageing. Biochimica Biophysica Acta (BBA) - Bioenergetics 1797, 961–967. 10.1016/j.bbabio.2010.01.004 20064485

[B12] Burdeos G. C. Nakagawa K. Kimura F. Miyazawa T. (2012). Tocotrienol attenuates triglyceride accumulation in HepG2 cells and F344 rats. Lipids 47, 471–481. 10.1007/s11745-012-3659-0 22367056

[B13] Calles-Escandón J. Poehlman E. T. (1997). Aging, fat oxidation and exercise. Aging (Milano) 9, 57–63. 10.1007/BF03340128 9177586

[B14] Ceci R. Duranti G. Leonetti A. Pietropaoli S. Spinozzi F. Marcocci L. (2017). Adaptive responses of heart and skeletal muscle to spermine oxidase overexpression: Evaluation of a new transgenic mouse model. Free Radic. Biol. Med. 103, 216–225. 10.1016/j.freeradbiomed.2016.12.040 28043891

[B15] Chai R. J. Vukovic J. Dunlop S. Grounds M. D. Shavlakadze T. (2011). Striking denervation of neuromuscular junctions without lumbar motoneuron loss in geriatric mouse muscle. PLOS ONE 6, e28090. 10.1371/journal.pone.0028090 22164231 PMC3229526

[B16] Chin K. Y. Wong S. K. Japar Sidik F. Z. Abdul Hamid J. Abas N. H. Mohd Ramli E. S. (2019). The effects of annatto tocotrienol supplementation on cartilage and subchondral bone in an animal model of osteoarthritis induced by monosodium iodoacetate. Int. J. Environ. Res. Public Health 16. 10.3390/ijerph16162897 PMC6720523 31412648

[B17] Chung E. Elmassry M. M. Kottapalli P. Kottapalli K. R. Kaur G. Dufour J. M. (2020). Metabolic benefits of annatto-extracted tocotrienol on glucose homeostasis, inflammation, and gut microbiome. Nutr. Res. 77, 97–107. 10.1016/j.nutres.2020.04.001 32438021

[B18] Chung E. Mo H. Wang S. Zu Y. Elfakhani M. Rios S. R. (2018). Potential roles of vitamin E in age-related changes in skeletal muscle health. Nutr. Res. 49, 23–36. 10.1016/j.nutres.2017.09.005 29420990

[B19] Clark B. C. Manini T. M. (2012). What is dynapenia? Nutrition 28, 495–503. 10.1016/j.nut.2011.12.002 22469110 PMC3571692

[B20] Consitt Le A. Dudley C. Saxena G. (2019). Impact of endurance and resistance training on skeletal muscle glucose metabolism in older adults. Nutrients 11, 2636. 10.3390/nu11112636 31684154 PMC6893763

[B21] Cooper C. Fielding R. Visser M. van Loon L. J. Rolland Y. Orwoll E. (2013). Tools in the assessment of sarcopenia. Calcif. Tissue Int. 93, 201–210. 10.1007/s00223-013-9757-z 23842964 PMC3744387

[B22] Cruz-Jentoft A. J. Baeyens J. Bauer J. P. Boirie J. M. Cederholm Y. Landi T. (2010). Sarcopenia: European consensus on definition and diagnosis: Report of the European working group on sarcopenia in older people. Age Ageing 39, 412–423. 10.1093/ageing/afq034 20392703 PMC2886201

[B23] Distefano Giovanna Goodpaster B. H. (2017). Effects of exercise and aging on skeletal muscle. Cold Spring Harb. Perspect. Med. 8, a029785. 10.1101/cshperspect.a029785 PMC5830901 28432116

[B24] Ermini M. (1976). Ageing changes in mammalian skeletal muscle. Gerontology 22, 301–316. 10.1159/000212145 131747

[B25] Fazelzadeh P. Hangelbroek R. W. Tieland M. de Groot L. C. Verdijk L. B. van Loon L. J. (2016). The muscle metabolome differs between healthy and frail older adults. J. Proteome Res. 15, 499–509. 10.1021/acs.jproteome.5b00840 26732810

[B26] Feng Z. Hanson R. W. Berger N. A. Trubitsyn T. (2016). Reprogramming of energy metabolism as a driver of aging. Oncotarget 7, 15410–15420. 10.18632/oncotarget.7645 26919253 PMC4941250

[B27] Frega N. Mozzon M. Bocci F. (1998). Identification and estimation of tocotrienols in the annatto lipid fraction by gas chromatography-mass spectrometry. J. Amer Oil Chem. Soc. 75, 1723–1727. 10.1007/s11746-998-0323-1

[B28] Fukui K. (2019). Neuroprotective and anti-obesity effects of tocotrienols. J. Nutr. Sci. Vitaminol. 65, S185–S187. 10.3177/jnsv.65.s185 31619626

[B29] Garvey S. M. Dugle J. E. Kennedy A. D. McDunn J. E. Kline W. Guo L. (2014). Metabolomic profiling reveals severe skeletal muscle group-specific perturbations of metabolism in aged FBN rats. Biogerontology 15, 217–232. 10.1007/s10522-014-9492-5 24652515 PMC4019835

[B30] Gasior K. Huber M. Lamura G. Lelkes O. Marin B. Rodrigues R. (2012). “Facts and figures on health ageing and long-term care,” in European Centre for social welfare policy and research (Vienna.

[B31] Gnoni A. Longo S. Gnoni G. V. Giudetti A. M. (2020). Carnitine in human muscle bioenergetics: Can carnitine supplementation improve physical exercise? Molecules 25, 25. 10.3390/molecules25010182 PMC6982879 31906370

[B32] Goodpaster B. H. Park S. W. Harris T. B. Kritchevsky S. B. Nevitt M. Schwartz A. V. (2006). The loss of skeletal muscle strength, mass, and quality in older adults: The health, aging and body composition study. Journals Gerontology Ser. A Biol. Sci. Med. Sci. 61, 1059–1064. 10.1093/gerona/61.10.1059 17077199

[B33] Goon D. E. Ab-Rahim S. Mohd Sakri A. H. Mohd Sakri M. Mazlan J. K. Tan M. (2021). 'Untargeted serum metabolites profiling in high-fat diet mice supplemented with enhanced palm tocotrienol-rich fraction using UHPLC-MS. Sci. Rep. 11, 21001. 10.1038/s41598-021-00454-9 34697380 PMC8546078

[B34] Goon Efendy Sheikh Abdul Kadir Siti Latip Normala Ab Ab. Rahim Sharaniza Ab Mazlan Musalmah (2019). Palm oil in lipid-based formulations and drug delivery systems. Biomolecules 9, 64. 10.3390/biom9020064 30781901 PMC6406477

[B35] Haleagrahara N. Swaminathan M. Chakravarthi S. Radhakrishnan A. (2014). 'Therapeutic efficacy of vitamin E δ-tocotrienol in collagen-induced rat model of arthritis. Biomed. Res. Int., 539540. 10.1155/2014/539540 25114906 PMC4119727

[B36] Hamasaki N. Hardjono I. S. Minakami S. (1978). 'Transport of phosphoenolpyruvate through the erythrocyte membrane. Biochem. J. 170, 39–46. 10.1042/bj1700039 629781 PMC1183858

[B37] He L. Li H. Wu A. Peng Y. Shu G. Yin G. (2019). 'Functions of N6-methyladenosine and its role in cancer. Mol. Cancer 18, 176. 10.1186/s12943-019-1109-9 31801551 PMC6892141

[B38] Horgan R. P. Kenny L. C. (2011). '‘Omic’ technologies: Genomics, transcriptomics, proteomics and metabolomics. Obstetrician Gynaecol. 13, 189–195. 10.1576/toag.13.3.189.27672

[B39] Houtkooper R. H. Argmann C. Houten S. M. Cantó C. Jeninga E. H. Andreux P. A. (2011). The metabolic footprint of aging in mice. Sci. Rep. 1, 134. 10.1038/srep00134 22355651 PMC3216615

[B40] Kameda M. Teruya T. Yanagida M. Kondoh H. (2020). 'Frailty markers comprise blood metabolites involved in antioxidation, cognition, and mobility. Proc. Natl. Acad. Sci. 117, 9483–9489. 10.1073/pnas.1920795117 32295884 PMC7196897

[B41] Kennedy B. K. Berger S. L. Brunet A. Campisi J. Cuervo A. M. Epel E. S. (2014). 'Geroscience: Linking aging to chronic disease. Cell. 159, 709–713. 10.1016/j.cell.2014.10.039 25417146 PMC4852871

[B42] Khor S. C. Abdul Karim N. Wan Ngah W. Z. Mohd Yusof Y. A. Makpol S. (2014). 'Vitamin E in sarcopenia: Current evidences on its role in prevention and treatment. Oxidative Med. Cell. Longev., 914853–53. 10.1155/2014/914853 PMC4109111 25097722

[B43] Khor S. C. Razak A. M. Wan Ngah W. Z. Mohd Yusof Y. A. Abdul Karim N. Makpol S. (2016). The tocotrienol-rich fraction is superior to tocopherol in promoting myogenic differentiation in the prevention of replicative senescence of myoblasts. PLOS ONE 11, e0149265. 10.1371/journal.pone.0149265 26885980 PMC4757569

[B44] Kob R. Fellner C. Bertsch T. Wittmann A. Mishura D. Sieber C. C. (2015). 'Gender-specific differences in the development of sarcopenia in the rodent model of the ageing high-fat rat. J. cachexia, sarcopenia muscle 6, 181–191. 10.1002/jcsm.12019 26136194 PMC4458084

[B45] Kohrt W. M. Holloszy J. O. (1995). 'Loss of skeletal muscle mass with aging: Effect on glucose tolerance. J. Gerontol. A Biol. Sci. Med. Sci. 50, 68–72. 10.1093/gerona/50a.special_issue.68 7493222

[B46] Lancha A. H. Jr. Poortmans J. R. Pereira L. O. (2009). 'The effect of 5 days of aspartate and asparagine supplementation on glucose transport activity in rat muscle. Cell. Biochem. Funct. 27, 552–557. 10.1002/cbf.1606 19821260

[B47] Lauretani F. Russo C. R. Bandinelli S. Bartali B. Cavazzini C. Di Iorio A. (2003). 'Age-associated changes in skeletal muscles and their effect on mobility: An operational diagnosis of sarcopenia. J. Appl. Physiol. 95, 1851–1860. 10.1152/japplphysiol.00246.2003 14555665

[B48] Lim J. J. Wan Zurinah W. N. Vincent M. Norwahidah A. K. (2019). Tocotrienol-rich fraction (TRF) treatment promotes proliferation capacity of stress-induced premature senescence myoblasts and modulates the renewal of satellite cells: Microarray analysis. Oxidative Med. Cell. Longev., 9141343. 10.1155/2019/9141343 PMC6350575 30774750

[B49] Liu X. Qu H. Zheng Y. Liao Q. Zhang L. Liao X. (2018). Mitochondrial glycerol 3-phosphate dehydrogenase promotes skeletal muscle regeneration. EMBO Mol. Med. 10. 10.15252/emmm.201809390 PMC6284384 30389681

[B50] Lo C-J. Ko Y-S. Chang S-W. Tang H-Y. Huang C-Y. Huang Y-C. (2020). 'Metabolic signatures of muscle mass loss in an elderly Taiwanese population. Aging 13, 944–956. 10.18632/aging.202209 33410783 PMC7834982

[B51] López-Otín C. Blasco M. A. Partridge L. Serrano M. Kroemer G. (2013). The hallmarks of aging. Cell. 153, 1194–1217. 23746838 10.1016/j.cell.2013.05.039 PMC3836174

[B52] Mahjabeen W. Khan D. A. Mirza S. A. Pervez M. A. (2021). 'Effects of delta-tocotrienol supplementation on glycemic control, oxidative stress, inflammatory biomarkers and miRNA expression in type 2 diabetes mellitus: A randomized control trial. Phytother. Res. 35, 3968–3976. 10.1002/ptr.7113 33899292

[B53] Mai M. Tönjes A. Kovacs P. Michael S. Fiedler G. M. Leichtle A. B. (2013). Serum levels of acylcarnitines are altered in prediabetic conditions. PLOS ONE 8, e82459. 10.1371/journal.pone.0082459 24358186 PMC3865089

[B54] Massudi H. Grant R. Braidy N. Guest J. Farnsworth B. Guillemin G. J. (2012). 'Age-associated changes in oxidative stress and NAD+ metabolism in human tissue. PLOS ONE 7, e42357. 10.1371/journal.pone.0042357 22848760 PMC3407129

[B55] Meredith C. N. Frontera W. R. Fisher E. C. Hughes V. A. Herland J. C. Edwards J. (1989). 'Peripheral effects of endurance training in young and old subjects. J. Appl. Physiol. 66, 2844–2849. 10.1152/jappl.1989.66.6.2844 2745349

[B56] Miljkovic N. Lim J. Y. Miljkovic I. Frontera W. R. (2015). Aging of skeletal muscle fibers. Ann. rehabilitation Med. 39 (2), 155–162. 10.5535/arm.2015.39.2.155 PMC4414960 25932410

[B57] Nemutlu E. Gupta A. Zhang S. Viqar M. Andre T. Jahangir A. (2015). Decline of phosphotransfer and substrate supply metabolic circuits hinders ATP cycling in aging myocardium. PLOS ONE 10, e0136556. 10.1371/journal.pone.0136556 26378442 PMC4574965

[B58] Newman A. B. Kupelian V. Visser M. Simonsick E. M. Goodpaster B. H. Kritchevsky S. B. (2006). 'Strength, but not muscle mass, is associated with mortality in the health, aging and body composition study cohort. J. Gerontol. A Biol. Sci. Med. Sci. 61, 72–77. 10.1093/gerona/61.1.72 16456196

[B59] Nichols S. O'Doherty A. F. Taylor C. Clark A. L. Carroll S. Ingle L. (2019). 'Low skeletal muscle mass is associated with low aerobic capacity and increased mortality risk in patients with coronary heart disease - a CARE CR study. Clin. Physiol. Funct. Imaging 39, 93–102. 10.1111/cpf.12539 30168241 PMC7379590

[B60] Obrador E. Salvador-Palmer R. Dellinger R. W. Estrela J. M. (2021). 'NAD(+) precursors and antioxidants for the treatment of amyotrophic lateral sclerosis. Biomedicines 9, 1000. 10.3390/biomedicines9081000 34440204 PMC8394119

[B61] Ortman J. M. Velkoff V. A. Hogan H. 2014. "An aging nation: The older population in the United States " In, 1–28.

[B62] Pang K. L. Chin K. Y. (2019). The Role of Tocotrienol in Protecting Against Metabolic Diseases, 24, 10.3390/molecules24050923 Molecules PMC6429133 30845769

[B63] Pataky M. Kumar A. Klaus K. Nair K. (2021). '498-P: Resistance exercise rapidly alters citric acid cycle and amino acid metabolism in skeletal muscle. Diabetes 70, 498. 10.2337/db21-498-p

[B64] Peh H. Y. Tan W. S. Liao W. Wong W. S. (2016). 'Vitamin E therapy beyond cancer: Tocopherol versus tocotrienol. Pharmacol. Ther. 162, 152–169. 10.1016/j.pharmthera.2015.12.003 26706242

[B65] Pehleman T. L. Peters S. J. Heigenhauser G. J. Spriet L. L. (2005). 'Enzymatic regulation of glucose disposal in human skeletal muscle after a high-fat, low-carbohydrate diet. J. Appl. Physiol. 98, 100–107. 10.1152/japplphysiol.00686.2004 15310747

[B66] Pötsch M. S. Tschirner A. Palus S. von Haehling S. Doehner W. Beadle J. (2014). The anabolic catabolic transforming agent (ACTA) espindolol increases muscle mass and decreases fat mass in old rats. J. cachexia, sarcopenia muscle 5, 149–158. 24272787 10.1007/s13539-013-0125-7 PMC4053568

[B67] Poulsen P. Wojtaszewski J. F. Petersen I. Christensen K. Richter E. A. Beck-Nielsen H. (2005). Impact of genetic versus environmental factors on the control of muscle glycogen synthase activation in twins. Diabetes 54, 1289–1296. 10.2337/diabetes.54.5.1289 15855312

[B68] Radhakrishnan A. Tudawe D. Chakravarthi S. Chiew G. S. Haleagrahara N. (2014). 'Effect of γ-tocotrienol in counteracting oxidative stress and joint damage in collagen-induced arthritis in rats. Exp. Ther. Med. 7, 1408–1414. 10.3892/etm.2014.1592 24940448 PMC3991526

[B69] Ramanathan N. Tan E. Loh L. J. Soh B. S. Yap W. N. (2018). 'Tocotrienol is a cardioprotective agent against ageing-associated cardiovascular disease and its associated morbidities. Nutr. Metab. (Lond) 15, 6. 10.1186/s12986-018-0244-4 29387138 PMC5775572

[B70] Ramos-Roman M. A. Sweetman L. Valdez M. J. Parks E. J. (2012). 'Postprandial changes in plasma acylcarnitine concentrations as markers of fatty acid flux in overweight and obesity. Metabolism 61, 202–212. 10.1016/j.metabol.2011.06.008 21820684 PMC3219812

[B71] Reginster J-Y. Cooper C. Rizzoli R. Kanis J. A. Appelboom G. Bautmans Ivan (2016). 'Recommendations for the conduct of clinical trials for drugs to treat or prevent sarcopenia. Aging - Clin. Exp. Res. 28, 47–58. 10.1007/s40520-015-0517-y 26717937 PMC4768478

[B72] Roberts A. W. Stellaogunwole U. Laura B. And Meganrabe A. (2018). “The population 65 Years and older in the United States,” in census.gov.

[B73] Roubenoff R. (2000). 'Sarcopenia and its implications for the elderly. Eur. J. Clin. Nutr. 54 (3), S40–S47. 10.1038/sj.ejcn.1601024 11041074

[B74] Schiaffino S. Mammucari C. (2011). Regulation of skeletal muscle growth by the IGF1-akt/PKB pathway: Insights from genetic models. Skelet. muscle 1, 4. 10.1186/2044-5040-1-4 21798082 PMC3143906

[B75] Schneider C. (2005). Chemistry and biology of vitamin E. Mol. Nutr. Food Res. 49, 7–30. 10.1002/mnfr.200400049 15580660

[B76] Sen C. K. Rink C. Khanna S. (2010). Palm oil-derived natural vitamin E alpha-tocotrienol in brain health and disease. J. Am. Coll. Nutr. 29 (3), 314s–323s. 10.1080/07315724.2010.10719846 20823491 PMC3065441

[B77] Shavlakadze T. Grounds M. (2006). 'Of bears, frogs, meat, mice and men: Complexity of factors affecting skeletal muscle mass and fat. Bioessays 28, 994–1009. 10.1002/bies.20479 16998828

[B78] Shen C. L. Klein A. Chin K. Y. Mo H. Tsai P. Yang R. S. (2017). 'Tocotrienols for bone health: A translational approach. Ann. N. Y. Acad. Sci. 1401, 150–165. 10.1111/nyas.13449 28891093

[B79] Sood S. Gallagher I. J. Lunnon K. Rullman E. Keohane A. Crossland H. (2015). 'A novel multi-tissue RNA diagnostic of healthy ageing relates to cognitive health status. Genome Biol. 16, 185. 10.1186/s13059-015-0750-x 26343147 PMC4561473

[B80] Sousa-Victor P. Gutarra S. García-Prat L. Rodriguez-Ubreva J. Ortet L. Ruiz-Bonilla V. (2014). 'Geriatric muscle stem cells switch reversible quiescence into senescence. Nature 506, 316–321. 10.1038/nature13013 24522534

[B81] Tarantini S. Yabluchanskiy A. Fülöp G. A. Kiss T. Perz A. O'Connor D. (2019). 'Age-Related alterations in gait function in freely moving male C57bl/6 mice: Translational relevance of decreased cadence and increased gait variability. J. Gerontol. A Biol. Sci. Med. Sci. 74, 1417–1421. 10.1093/gerona/gly242 30383221 PMC6696715

[B82] van den Berg Robert A. HuubHoefslootSmilde C. J. Johan A. Westerhuis, Age K. van der Werf Mariët J. (2006). 'Centering, scaling, and transformations: Improving the biological information content of metabolomics data. BMC Genomics 7, 142. 10.1186/1471-2164-7-142 16762068 PMC1534033

[B83] Wang Y. Zeng L. Liang C. Zan R. Ji W. Zhang Z. (2019). 'Integrated analysis of transcriptome-wide m(6)A methylome of osteosarcoma stem cells enriched by chemotherapy. Epigenomics 11, 1693–1715. 10.2217/epi-2019-0262 31650864

[B84] Xia J. Broadhurst D. I. Wilson M. Wishart D. S. (2013). Translational biomarker discovery in clinical metabolomics: An introductory tutorial. Metabolomics 9, 280–299. 10.1007/s11306-012-0482-9 23543913 PMC3608878

[B85] Xia J. Wishart D. S. (2016). 'Using MetaboAnalyst 3.0 for comprehensive metabolomics data analysis. Curr. Protoc. Bioinforma. 55, 14. 10.1-14.10.9110.1002/cpbi.11 27603023

[B86] Xiang C. Zhang Y. Chen Q. Sun A. Peng Y. Zhang G. (2021). 'Increased glycolysis in skeletal muscle coordinates with adipose tissue in systemic metabolic homeostasis. J. Cell. Mol. Med. 25, 7840–7854. 10.1111/jcmm.16698 34227742 PMC8358859

[B87] Yuan Y. Hakimi P. Kao C. Kao A. Liu R. Janocha A. (2016). 'Reciprocal changes in phosphoenolpyruvate carboxykinase and pyruvate kinase with age are a determinant of aging in *Caenorhabditis elegans* . J. Biol. Chem. 291, 1307–1319. 10.1074/jbc.m115.691766 26631730 PMC4714217

[B88] Zhang X. Yao Y. Han J. Yang Y. Chen Y. Tang Z. (2020). 'Longitudinal epitranscriptome profiling reveals the crucial role of N(6)-methyladenosine methylation in porcine prenatal skeletal muscle development. J. Genet. Genomics 47, 466–476. 10.1016/j.jgg.2020.07.003 33268291

[B89] Zhao L. Fang X. Marshall M. R. Chung S. (2016). Regulation of obesity and metabolic complications by gamma and delta tocotrienols. Molecules 21, 344. 10.3390/molecules21030344 26978344 PMC6274282

[B90] Zierer J. Menni C. Kastenmüller G. Spector T. D. (2015). 'Integration of 'omics' data in aging research: From biomarkers to systems biology. Aging Cell. 14, 933–944. 10.1111/acel.12386 26331998 PMC4693464

