# Erratum: Untargeted muscle tissue metabolites profiling in young, adult, and old rats supplemented with tocotrienol-rich fraction

**DOI:** 10.3389/fmolb.2022.1119445

**Published:** 2022-12-21

**Authors:** Frontiers Production Office

**Affiliations:** Frontiers Media SA, Lausanne, Switzerland

**Keywords:** tocotrienol, sarcopenia, untargeted metabolites, skeletal muscle, ageing

Due to a production error, the legends of Figures 2–8 were not implemented correctly.

**FIGURE 2:**
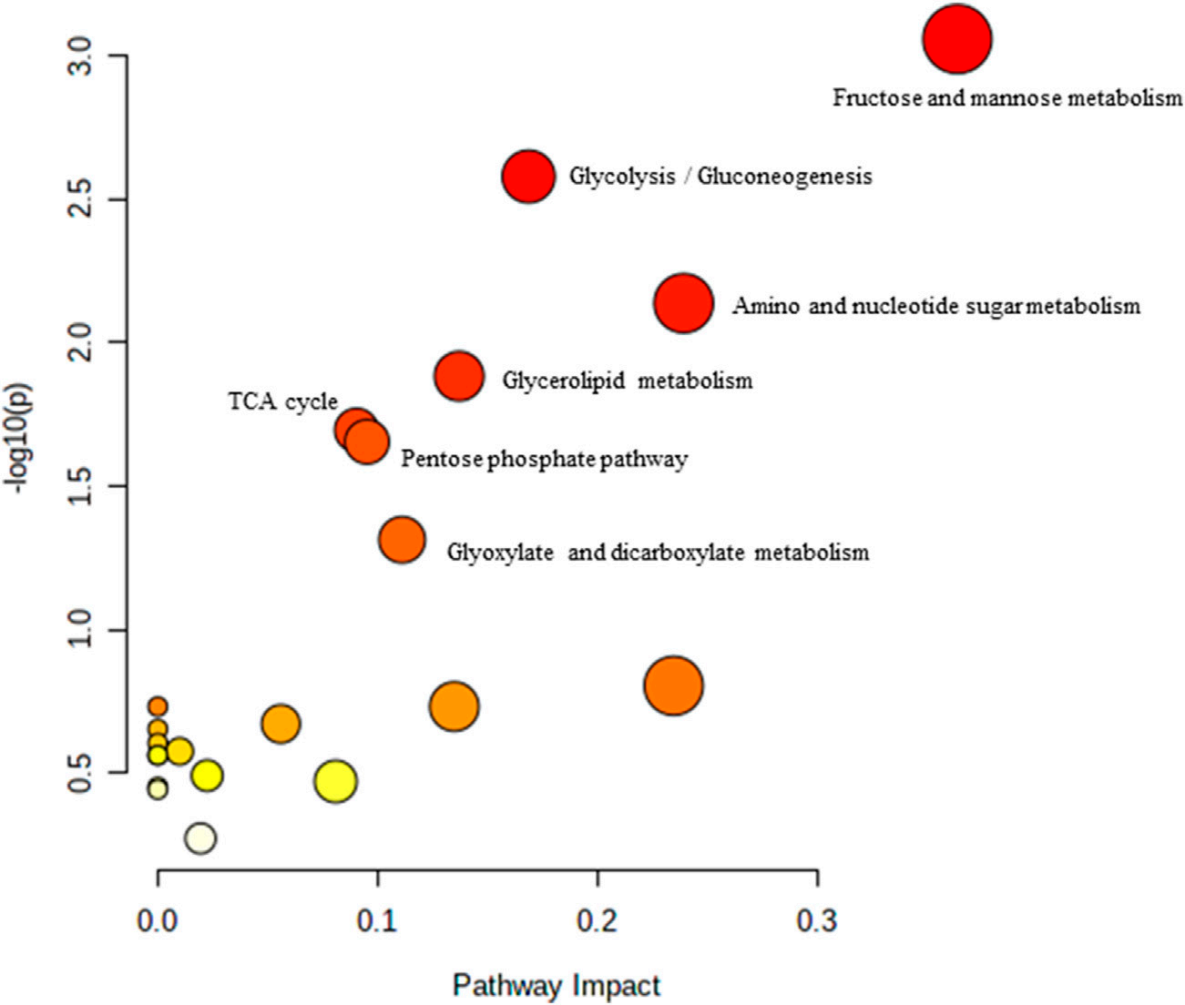
Biochemical pathway analysis of metabolites profiled for YC vs. AC.

**FIGURE 3:**
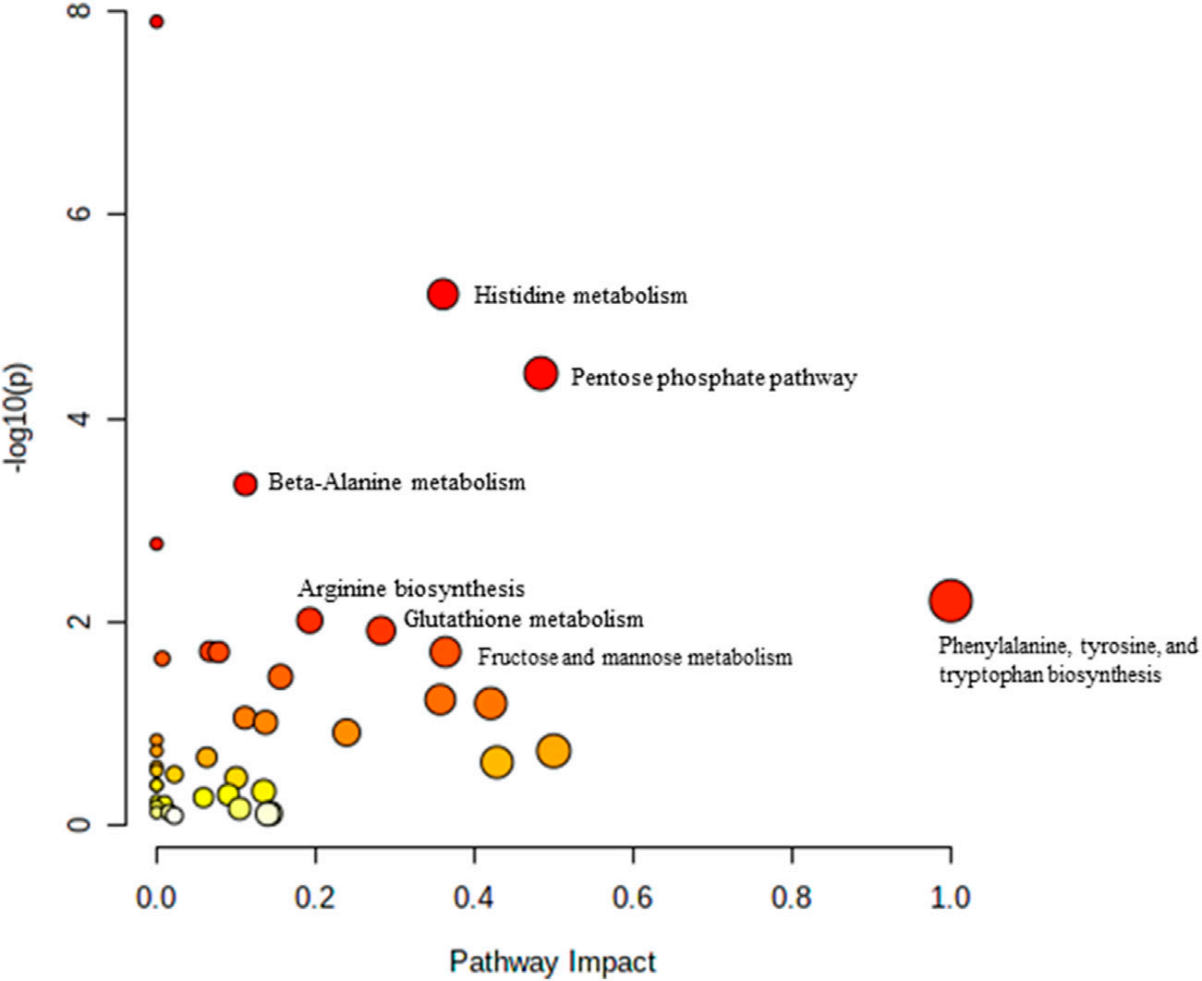
Biochemical pathway analysis of metabolites profiled for YC vs. OC.

**FIGURE 4:**
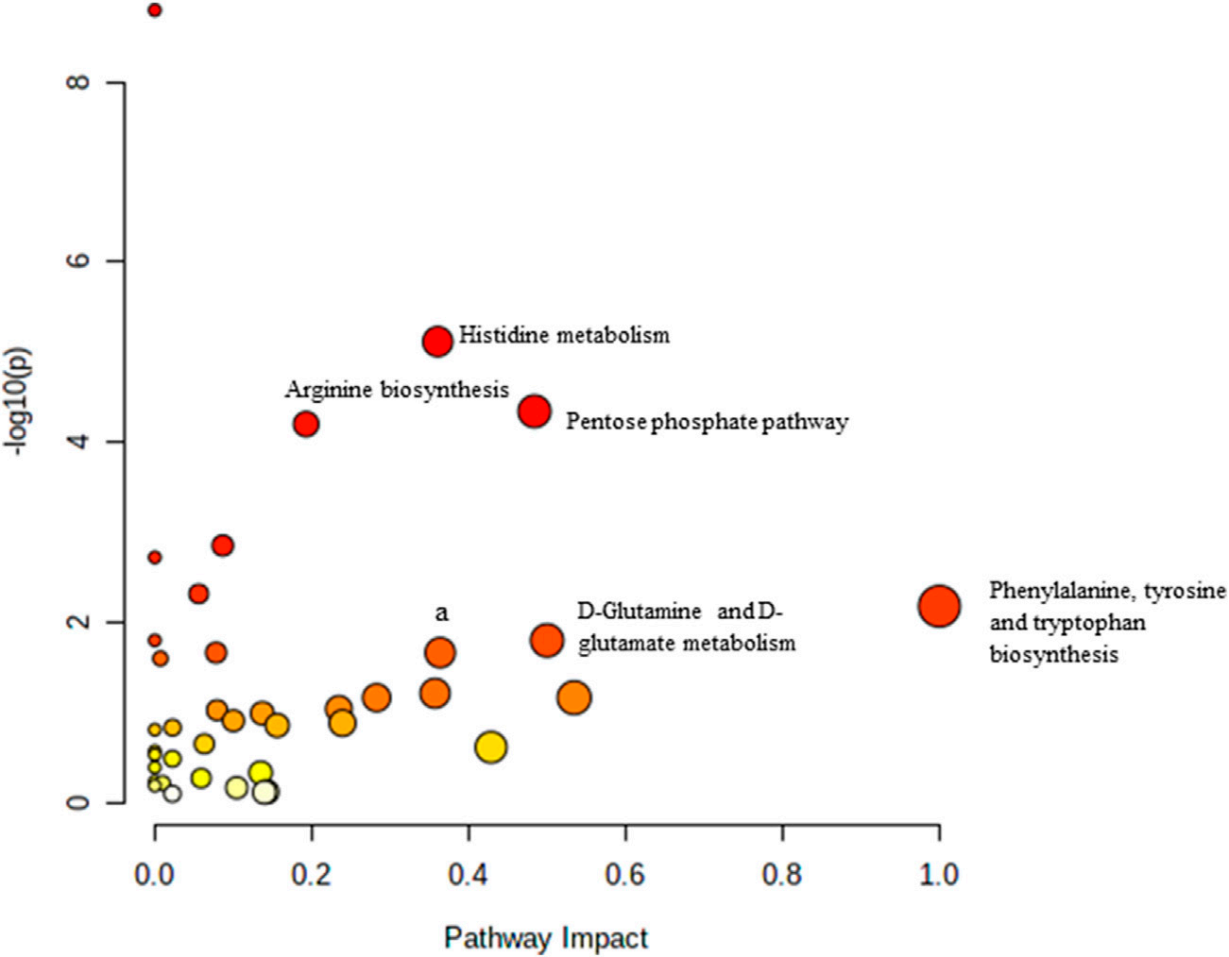
Biochemical pathway analysis of metabolites profiled for AC vs. OC. a: Fructose and mannose metabolism.

**FIGURE 5:**
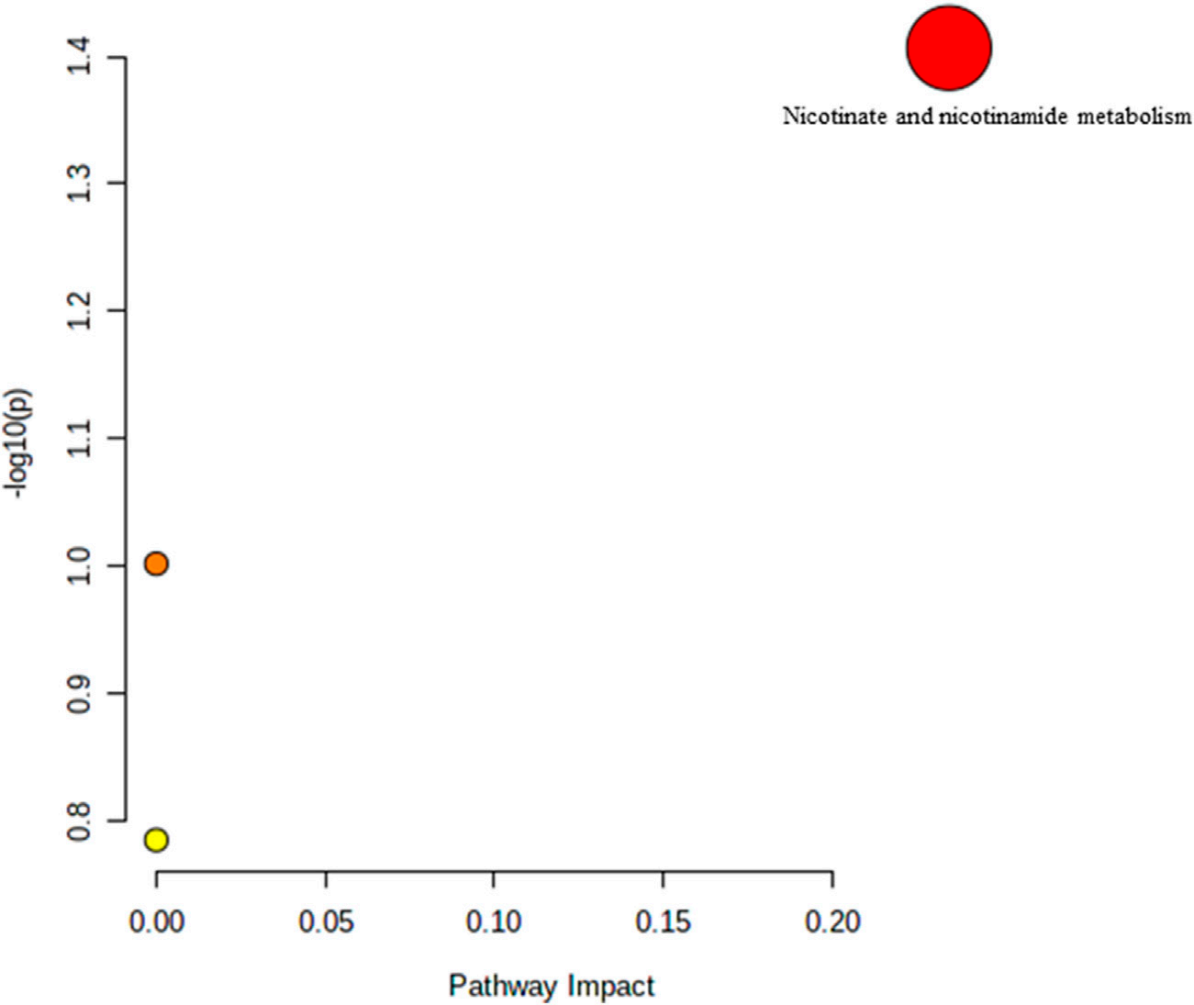
Biochemical pathway analysis of metabolites profiled for YC vs. YT.

**FIGURE 6:**
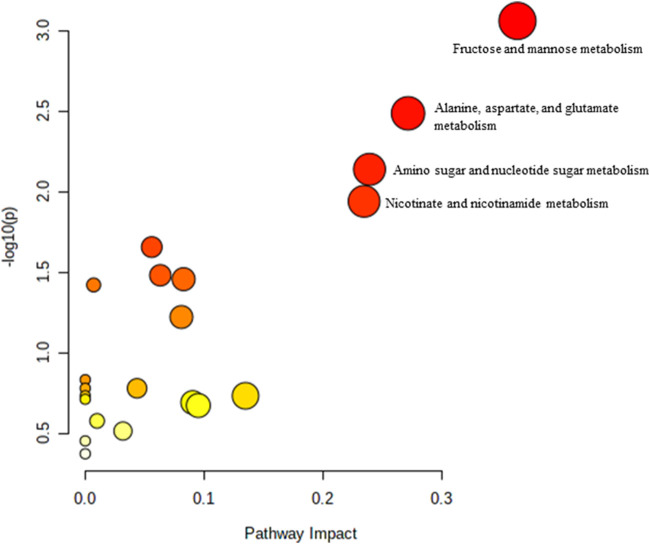
Biochemical pathway analysis of metabolites profiled for AC vs. AT.

**FIGURE 7:**
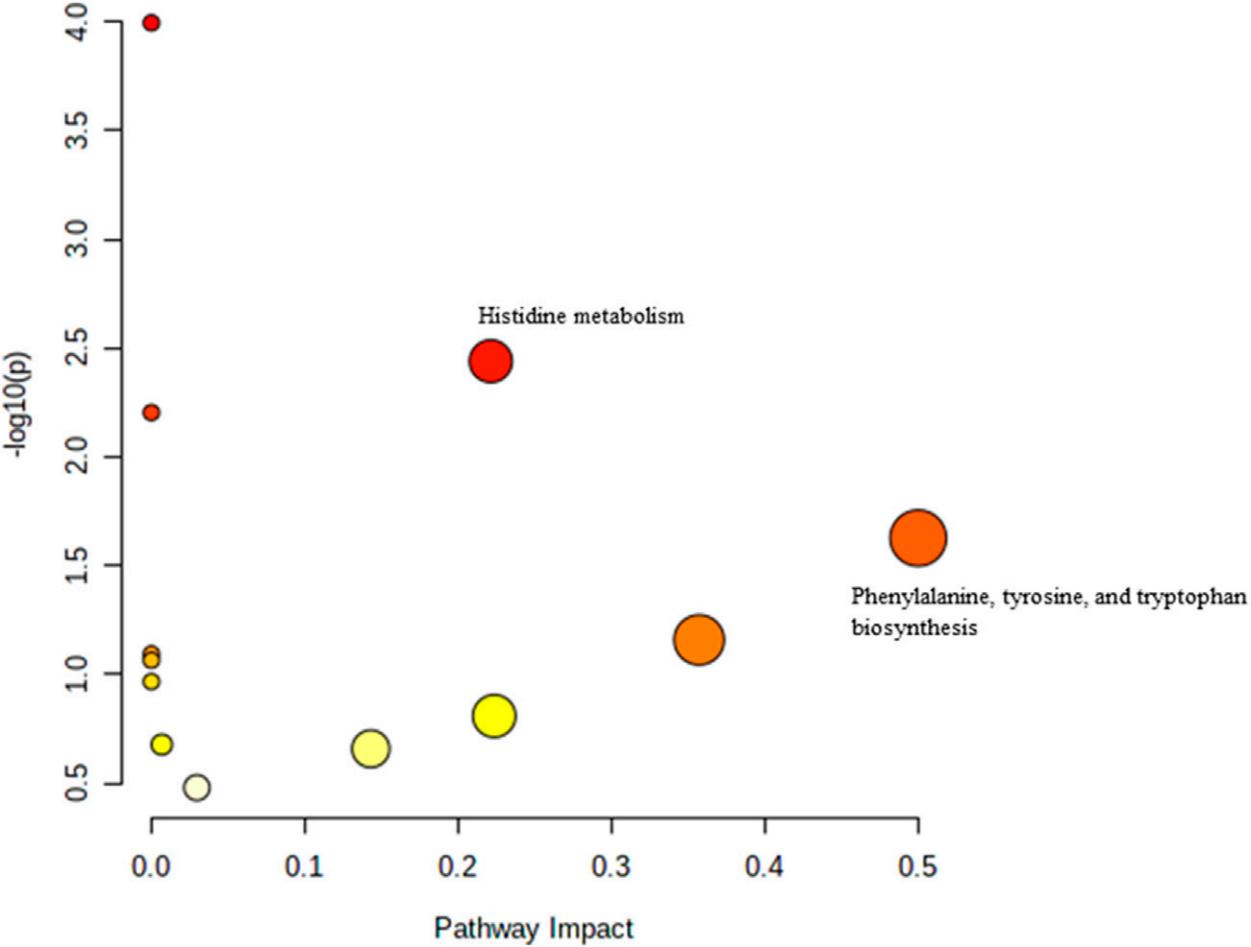
Biochemical pathway analysis of metabolites profiled for OC vs. OT.

**FIGURE 8:**
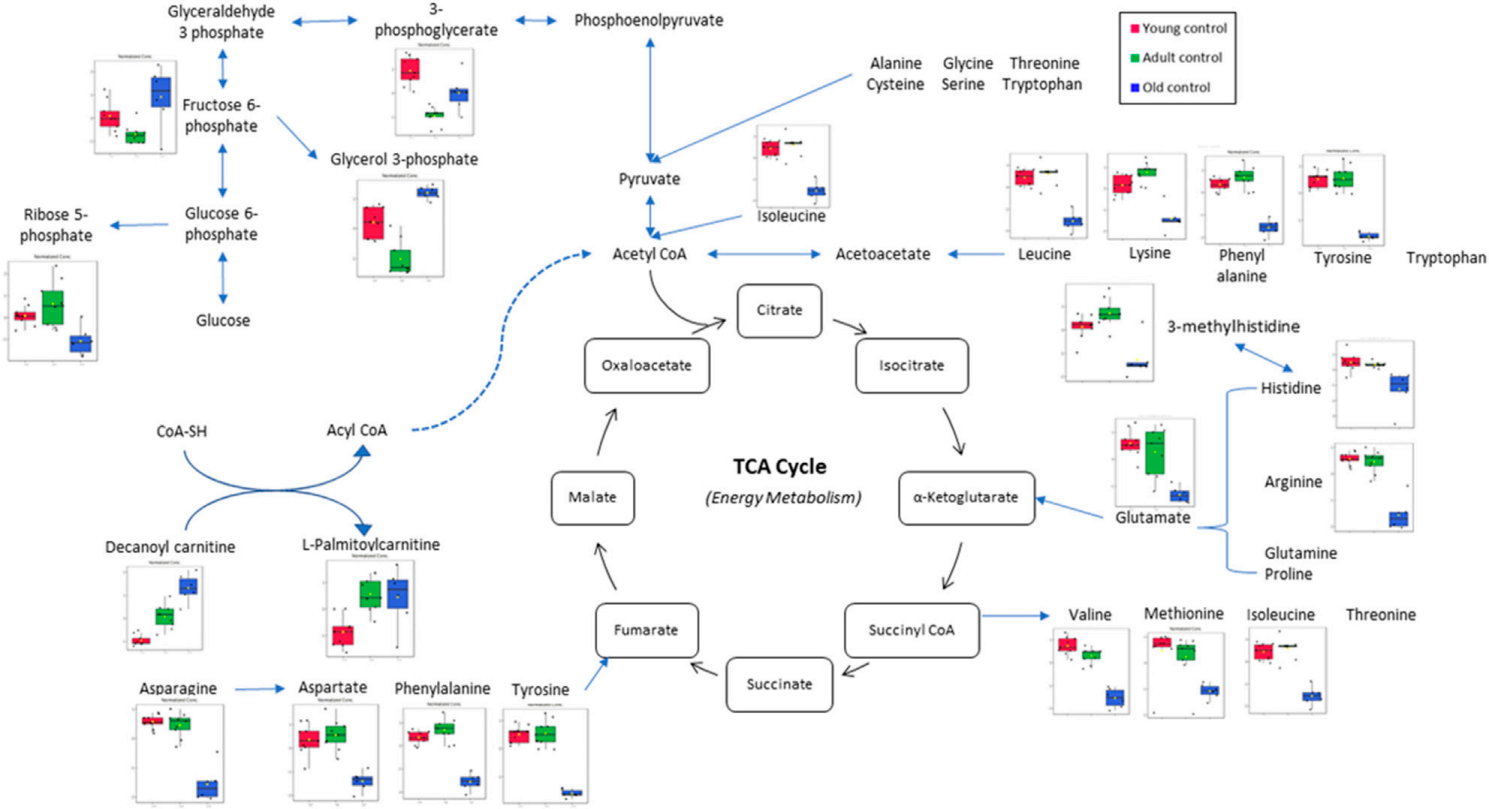
Muscle metabolomes changes in ageing rats. Metabolite concentrations in muscle specimens are depicted graphically and subjected to quantitative analysis. Box and whiskers plots with 95% confidence intervals are presented for quantified amino acids. Analysis was performed using one-way Anova with Tukey’s post hoc analysis. *p < 0.05.

Figure 2 Legend reads as “Biochemical pathway analysis of metabolites profiled for YC vs. OC. **(A)** Purine metabolism; **(B)** Pentose and glucuronate interconversions **(C)** Arginine and proline metabolism.” The correct legend for Figure 2 is “'Biochemical pathway analysis of metabolites profiled for YC vs. AC.”

Figure 3 Legend reads as “Biochemical pathway analysis of metabolites profiled for AC vs. OC. a: Fructose and mannose metabolism.” The correct legend for Figure 3 is “Biochemical pathway analysis of metabolites profiled for YC vs. OC”.

Figure 4 Legend reads as “Biochemical pathway analysis of metabolites profiled for YC vs. YT.” The correct legend for Figure 4 is “Biochemical pathway analysis of metabolites profiled for AC vs. OC. a: Fructose and mannose metabolism”.

Figure 5 Legend reads as “Biochemical pathway analysis of metabolites profiled for AC vs. AT.” The correct legend for Figure 5 is “Biochemical pathway analysis of metabolites profiled for YC vs. YT”.

Figure 6 Legend reads as “Biochemical pathway analysis of metabolites profiled for OC vs. OT.” The correct legend for Figure 6 is “Biochemical pathway analysis of metabolites profiled for AC vs. AT”.

Figure 7 Legend reads as “Muscle metabolomes changes in ageing rats. Metabolite concentrations in muscle specimens are depicted graphically and subjected to quantitative analysis. Box and whiskers plots with 95% confidence intervals are presented for quantified amino acids. Analysis was performed using one-way Anova with Tukey’s *post hoc* analysis. *p < 0.05.” The correct legend for Figure 7 is “Biochemical pathway analysis of metabolites profiled for OC vs. OT”.

Figure 8 Legend reads as “Significant metabolic changes after tocotrienol-rich fraction treatment.” The correct legend for Figure 8 is “Muscle metabolomes changes in ageing rats. Metabolite concentrations in muscle specimens are depicted graphically and subjected to quantitative analysis. Box and whiskers plots with 95% confidence intervals are presented for quantified amino acids. Analysis was performed using one-way Anova with Tukey’s *post hoc* analysis. *p < 0.05”.

The publisher apologizes for the mistake. The original article has been updated.

