# Supplementary material for: Untargeted muscle tissue metabolites profiling in young, adult, and old rats supplemented with tocotrienol-rich fraction
Source: Front Mol Biosci. 2022 Oct 14;9:1008908. doi: 10.3389/fmolb.2022.1008908 (PMC9616602; doi:10.3389/fmolb.2022.1008908)
Supplement: Supplementary file 1 [file DataSheet1.zip › Supp Table S1.docx]

| **Table S1:** List of biochemical pathways (MetaboAnalyst) identified for **YC vs AC** | | | |
| --- | --- | --- | --- |
| **Pathway** | **Match Status** | **p-value** | **Impact** |
| Fructose and mannose metabolism | 3/18 | 0.00* | 0.36^#^ |
| Glycolysis / Gluconeogenesis | 3/26 | 0.00* | 0.17^#^ |
| Amino sugar and nucleotide sugar metabolism | 3/37 | 0.01* | 0.24^#^ |
| Glycerolipid metabolism | 2/16 | 0.01* | 0.14^#^ |
| Citrate cycle (TCA cycle) | 2/20 | 0.02* | 0.10^#^ |
| Pentose phosphate pathway | 2/21 | 0.02* | 0.10^#^ |
| Glyoxylate and dicarboxylate metabolism | 2/32 | 0.05* | 0.11^#^ |
| Nicotinate and nicotinamide metabolism | 1/15 | 0.16 | 0.23 |
| Pentose and glucuronate interconversions | 1/18 | 0.19 | 0.0 |
| Starch and sucrose metabolism | 1/18 | 0.19 | 0.13 |
| Beta-Alanine metabolism | 1/21 | 0.21 | 0.06 |
| Pyruvate metabolism | 1/22 | 0.22 | 0.0 |
| Lysine degradation | 1/25 | 0.25 | 0.0 |
| Galactose metabolism | 1/27 | 0.27 | 0.01 |
| Alanine, aspartate and glutamate metabolism | 1/28 | 0.27 | 0.0 |
| Glutathione metabolism | 1/28 | 0.27 | 0.0 |
| Glycine, serine and threonine metabolism | 1/34 | 0.32 | 0.02 |
| Glycerophospholipid metabolism | 1/36 | 0.34 | 0.08 |
| Arginine and proline metabolism | 1/38 | 0.35 | 0.0 |
| Fatty acid degradation | 1/39 | 0.36 | 0.0 |
| Purine metabolism | 1/66 | 0.53 | 0.02 |
| *p-value <0.05; and ^#^impact > 0.1 is regard as significant. | | | |
