# Supplementary material for: Untargeted muscle tissue metabolites profiling in young, adult, and old rats supplemented with tocotrienol-rich fraction
Source: Front Mol Biosci. 2022 Oct 14;9:1008908. doi: 10.3389/fmolb.2022.1008908 (PMC9616602; doi:10.3389/fmolb.2022.1008908)
Supplement: Supplementary file 1 [file DataSheet1.zip › Supp Table S2.docx]

| **Table S2:** List of biochemical pathways (MetaboAnalyst) identified for **YC vs OC** | | | |
| --- | --- | --- | --- |
| **Pathway** | **Match Status** | **p-value** | **Impact** |
| Aminoacyl-tRNA biosynthesis | 12/48 | 0.00 | 0.0 |
| Histidine metabolism | 6/16 | 0.00* | 0.36^#^ |
| Pentose phosphate pathway | 6/21 | 0.00* | 0.48^#^ |
| Beta-Alanine metabolism | 5/21 | 0.00* | 0.11^#^ |
| Valine, leucine and isoleucine biosynthesis | 3/8 | 0.00 | 0.0 |
| Phenylalanine, tyrosine and tryptophan biosynthesis | 2/4 | 0.01* | 1.0# |
| Arginine biosynthesis | 3/14 | 0.01* | 0.19# |
| Glutathione metabolism | 4/28 | 0.01* | 0.28# |
| Purine metabolism | 6/66 | 0.02* | 0.10# |
| Pentose and glucuronate interconversions | 3/18 | 0.02* | 0.10# |
| Fructose and mannose metabolism | 3/18 | 0.02* | 0.36# |
| Pantothenate and CoA biosynthesis | 3/19 | 0.02* | 0.01 |
| Arginine and proline metabolism | 4/38 | 0.03* | 0.16# |
| Phenylalanine metabolism | 2/12 | 0.06 | 0.36 |
| Alanine, aspartate and glutamate metabolism | 3/28 | 0.06 | 0.42 |
| Glyoxylate and dicarboxylate metabolism | 3/32 | 0.09 | 0.11 |
| Glycerolipid metabolism | 2/16 | 0.10 | 0.14 |
| Amino sugar and nucleotide sugar metabolism | 3/37 | 0.12 | 0.24 |
| Valine, leucine and isoleucine degradation | 3/40 | 0.14 | 0.0 |
| Nitrogen metabolism | 1/6 | 0.18 | 0.0 |
| D-Glutamine and D-Glutamate metabolism | 1/6 | 0.18 | 0.5 |
| Glycolysis / Gluconeogenesis | 2/26 | 0.21 | 0.06 |
| Taurine and hypotaurine metabolism | 1/8 | 0.24 | 0.43 |
| Ubiquinone and other terpenoid-quinone biosynthesis | 1/9 | 0.26 | 0.0 |
| Biotin metabolism | 1/10 | 0.29 | 0.0 |
| Glycine, serine and threonine metabolism | 2/34 | 0.31 | 0.02 |
| Butanoate metabolism | 1/15 | 0.40 | 0.0 |
| Nicotinate and nicotinamide metabolism | 1/15 | 0.40 | 0.0 |
| Starch and sucrose metabolism | 1/18 | 0.46 | 0.13 |
| TCA cycle | 1/20 | 0.49 | 0.09 |
| Pyruvate metabolism | 1/22 | 0.53 | 0.06 |
| Lysine degradation | 1/25 | 0.57 | 0.0 |
| Galactose metabolism | 1/27 | 0.60 | 0.01 |
| Porphyrine and chlorophyll metabolism | 1/30 | 0.64 | 0.0 |
| Cysteine and methionine metabolism | 1/33 | 0.68 | 0.10 |
| Fatty acid degradation | 1/39 | 0.74 | 0.02 |
| Tryptophan metabolism | 1/41 | 0.75 | 0.14 |
| Tyrosine metabolism | 1/42 | 0.76 | 0.14 |
| Primary bile acid biosynthesis | 1/46 | 0.79 | 0.02 |
| *p-value <0.05; and ^#^impact > 0.1 is regard as significant. | | | |
