# Supplementary material for: Untargeted muscle tissue metabolites profiling in young, adult, and old rats supplemented with tocotrienol-rich fraction
Source: Front Mol Biosci. 2022 Oct 14;9:1008908. doi: 10.3389/fmolb.2022.1008908 (PMC9616602; doi:10.3389/fmolb.2022.1008908)
Supplement: Supplementary file 1 [file DataSheet1.zip › Supp Table S4.docx]

| **Table S4:** List of biochemical pathways (MetaboAnalyst) identified for **YC vs YT** | | | |
| --- | --- | --- | --- |
| **Pathway** | **Match Status** | **p-value** | **Impact** |
| Nicotinate and nicotinamide metabolism | 1/15 | 0.039* | 0.23465^#^ |
| Fatty acid degradation | 1/39 | 0.10 | 0.0 |
| Purine metabolism | 1/66 | 0.164 | 0.0 |
| *p-value <0.05; and ^#^impact > 0.1 is regard as significant. | | | |
