# Supplementary material for: Untargeted muscle tissue metabolites profiling in young, adult, and old rats supplemented with tocotrienol-rich fraction
Source: Front Mol Biosci. 2022 Oct 14;9:1008908. doi: 10.3389/fmolb.2022.1008908 (PMC9616602; doi:10.3389/fmolb.2022.1008908)
Supplement: Supplementary file 1 [file DataSheet1.zip › Supp Table S5.docx]

| **Table S5:** List of biochemical pathways (MetaboAnalyst) identified for **AC vs AT** | | | |
| --- | --- | --- | --- |
| **Pathway** | **Match Status** | **p-value** | **Impact** |
| Fructose and mannose metabolism | 3/18 | 0.00* | 0.36# |
| Alanine, aspartate and glutamate metabolism | 3/28 | 0.00* | 0.27# |
| Amino sugar and nucleotide sugar metabolism | 3/37 | 0.01* | 0.24# |
| Nicotinate and nicotinamide metabolism | 2/15 | 0.01* | 0.23# |
| Beta-Alanine metabolism | 2/21 | 0.02 | 0.06 |
| Glycolysis / Gluconeogenesis | 2/26 | 0.03 | 0.06 |
| Purine metabolism | 3/66 | 0.04 | 0.08 |
| Glutathione metabolism | 2/28 | 0.04 | 0.01 |
| Glycerophospholipid metabolism | 2/36 | 0.06 | 0.10 |
| Arginine biosynthesis | 1/14 | 0.15 | 0.0 |
| Histidine metabolism | 1/16 | 0.17 | 0.0 |
| Glycerolipid metabolism | 1/16 | 0.17 | 0.04 |
| Pentose and glucuronate interconversions | 1/18 | 0.19 | 0.13 |
| Starch and sucrose metabolism | 1/18 | 0.19 | 0.13 |
| Pantothenate and CoA biosynthesis | 1/19 | 0.19 | 0.0 |
| TCA cycle | 1/20 | 0.21 | 0.10 |
| Pentose phosphate pathway | 1/21 | 0.21 | 0.10 |
| Galactose metabolism | 1/27 | 0.27 | 0.01 |
| Glyoxylate and dicarboxylate metabolism | 1/32 | 0.31 | 0.03 |
| Arginine and proline metabolism | 1/38 | 0.35 | 0.0 |
| Aminoacyl-tRNA biosynthesis | 1/48 | 0.42 | 0.0 |
| *p-value <0.05; and ^#^impact > 0.1 is regard as significant. | | | |
