# Supplementary material for: Untargeted muscle tissue metabolites profiling in young, adult, and old rats supplemented with tocotrienol-rich fraction
Source: Front Mol Biosci. 2022 Oct 14;9:1008908. doi: 10.3389/fmolb.2022.1008908 (PMC9616602; doi:10.3389/fmolb.2022.1008908)
Supplement: Supplementary file 1 [file DataSheet1.zip › Supp Table S6.docx]

| **Table S6:** List of biochemical pathways (MetaboAnalyst) identified **for OC vs OT** | | | |
| --- | --- | --- | --- |
| **Pathway** | **Match Status** | **p-value** | **Impact** |
| Aminoacyl-tRNA biosynthesis | 4/48 | 0.00 | 0.0 |
| Histidine metabolism | 2/16 | 0.00* | 0.22# |
| Beta-Alanine metabolism | 2/21 | 0.01 | 0.0 |
| Phenylalanine, tyrosine and tryptophan biosynthesis | 1/4 | 0.02* | 0.5# |
| Phenylalanine metabolism | 1/12 | 0.07 | 0.36 |
| Arginine biosynthesis | 1/14 | 0.08 | 0.0 |
| Nicotinate and nicotinamide metabolism | 1/15 | 0.09 | 0.0 |
| Pantothenate and CoA biosynthesis | 1/19 | 0.11 | 0.0 |
| Alanine, aspartate and glutamate metabolism | 1/28 | 0.16 | 0.22 |
| Pyrimidine metabolism | 1/39 | 0.21 | 0.01 |
| Tryptophan metabolism | 1/41 | 0.22 | 0.14 |
| Purine metabolism | 1/66 | 0.33 | 0.03 |
| *p-value <0.05; and ^#^impact > 0.1 is regard as significant. | | | |
