# Supplementary figures and images for: Untargeted muscle tissue metabolites profiling in young, adult, and old rats supplemented with tocotrienol-rich fraction
Source: Front Mol Biosci. 2022 Oct 14;9:1008908. doi: 10.3389/fmolb.2022.1008908 (PMC9616602; doi:10.3389/fmolb.2022.1008908)

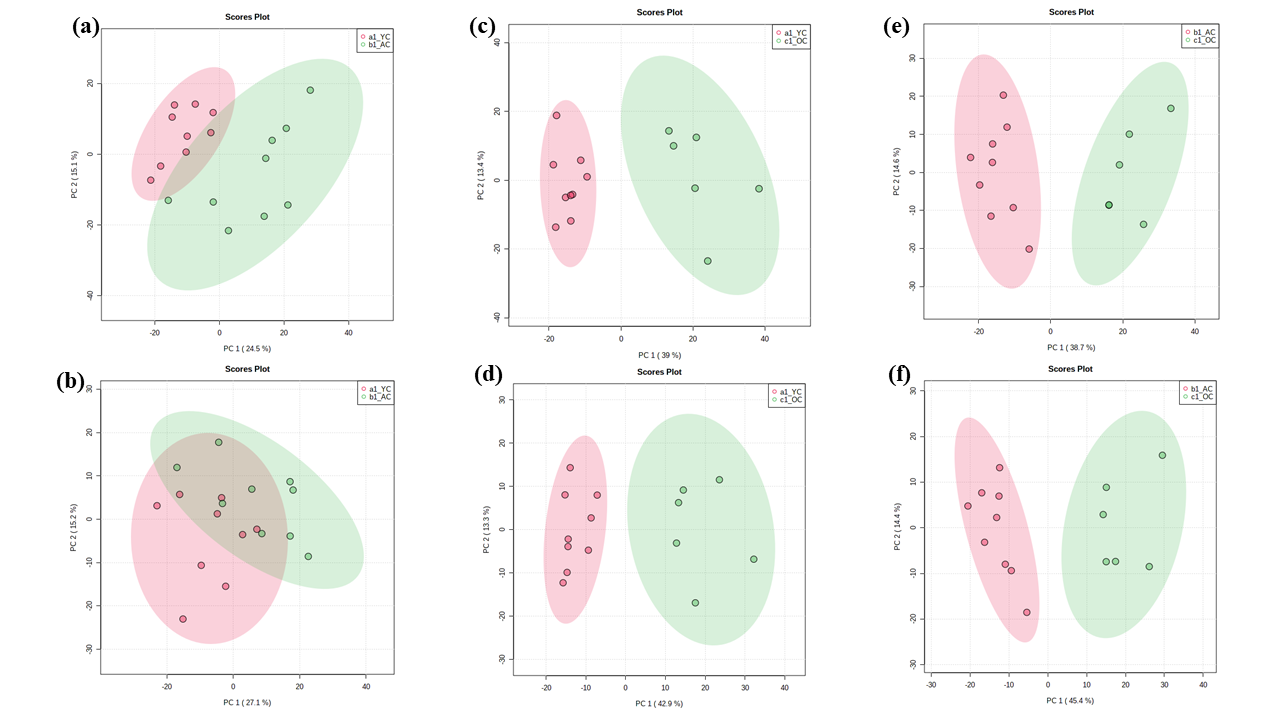

Supplement: Supplementary file 1 [file DataSheet1.zip › Supp Figure S1.tif]

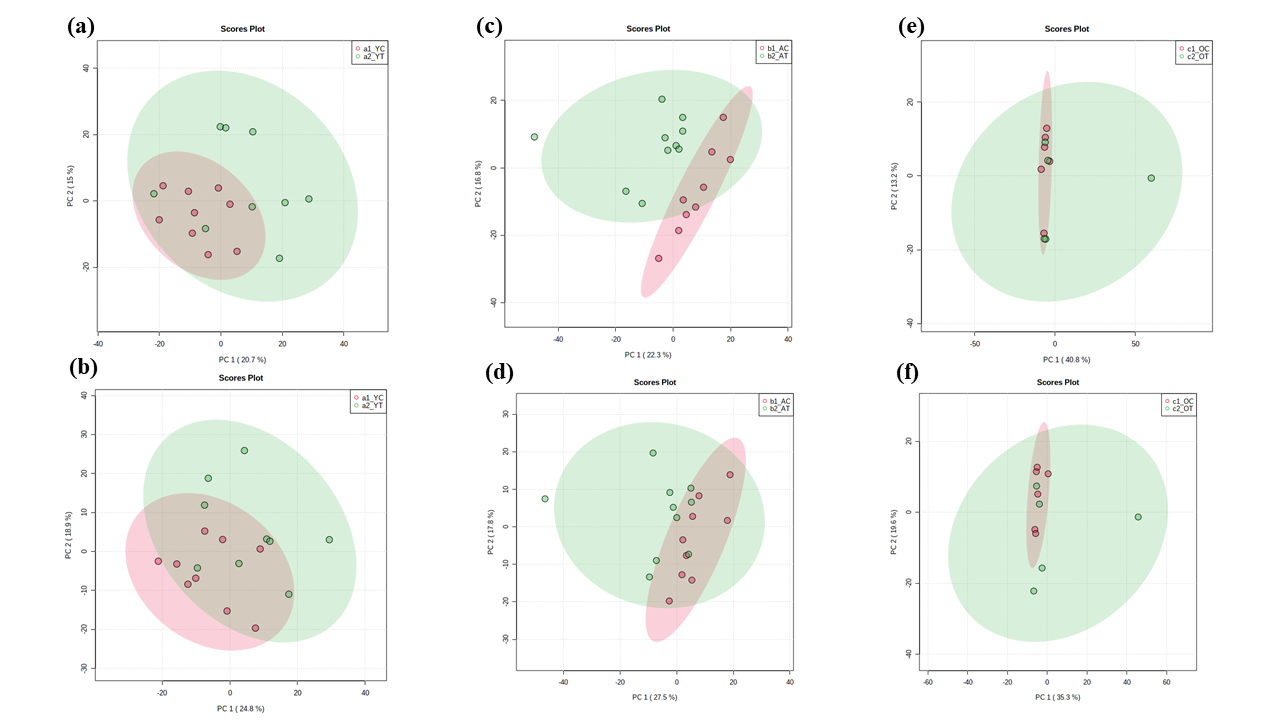

Supplement: Supplementary file 1 [file DataSheet1.zip › Supp Figure S2.tif]
